# Revealing the Improving Effect and Molecular Mechanism of *L*-Clausenamide in Combating the Acute Lung Injury: Insights from Network Pharmacology, Molecular Docking, and In Vitro Validation

**DOI:** 10.3390/biology14070836

**Published:** 2025-07-09

**Authors:** Yu Fu, Nannan Wang, Jinhai Luo, Yanyi Huang, Baoning Liu, Charles S. Brennan, Baojun Xu, Jincan Luo

**Affiliations:** 1 Guangzhou National Laboratory, International Bio-Island, Guangzhou 510005, China; fu_yu@gzlab.ac.cn (Y.F.); wnn285060315@126.com (N.W.); liu_baoning@gzlab.ac.cn (B.L.); 2 Food Science and Technology Program, Department of Life Sciences, Beijing Normal-Hong Kong Baptist University, Zhuhai 519087, China; luojinhai@uic.edu.cn; 3 School of Science, RMIT University, Bundoora West Campus, Plenty Road, Melbourne, VIC 3083, Australia; yanyi.huang@student.rmit.edu.au (Y.H.); charles.brennan@rmit.edu.au (C.S.B.); 4 Key Laboratory of Viral Pathogenesis & Infection Prevention and Control (Jinan University), Ministry of Education, Guangzhou 510632, China

**Keywords:** *L*-Clausenamide, acute lung injury, ROS, apoptosis, *Clausena lansium*

## Abstract

Acute lung injury is a severe disease with a high mortality rate, which can result in increased oxidative stress and further mitochondrial damage and cell apoptosis. *L*-Clausenamide is an amide from the fruit wampee. This study combined network pharmacology, molecular docking, and in vitro study to elucidate the effect of combating acute lung injury and the underlying mechanism of *L*-Clausenamide. Network pharmacology indicated that the 152 targets can treat acute lung injury through regulating oxidative stress. Based on PPI analysis and screening of the central target, AKT1 is the key target of the underlying mechanism. KEGG and GO enrichment analysis demonstrated that apoptosis is an important pathway for this curing effect. In the in vitro study, treatment with *L*-Clausenamide alleviates intracellular ROS accumulation, mitochondrial membrane potential loss, mitochondrial morphological distortion, ATP decrease, and the CASP3 activity. The SPR analysis was performed to validate the binding between AKT1 and *L*-Clausenamide. The Western blot result showed that *L*-Clausenamide increases the phosphorylation of Akt and decreases cleavage of CASP3. *L*-Clausenamide can alleviate lipopolysaccharide (LPS)-induced acute lung injury through targeting AKT1 and show an improvement in mitochondrial abnormality and inhibition against ROS-activated caspase-3-dependent apoptosis activation.

## Simple Summary

Acute lung injury (ALI) is a life-threatening disease, causing intrinsic classical apoptosis. Lipopolysaccharide (LPS) is commonly used to mimic ALI in vitro. *L*-Clausenamide is an amide from the fruit wampee. We found it is capable of alleviating LPS-mediated reactive oxygen species (ROS) and DNA damage accumulation, ATP decline, mitochondrial depolarization, and structural and functional collapse. By prediction and verification, we found both the AKT1 protein and Caspase-3 play an important role in the alleviation effect of *L*-Clausenamide. This finding reveals *L*-Clausenamide has developmental potential for ALI treatment, and further study on this mechanism may discover the effect among *L*-Clausenamide, AKT1, and Caspase-3.

## 1. Introduction

Acute lung injury (ALI) is a severe disease with a high mortality rate, characterized by inflammation, endothelial and epithelial barrier damage, and gas exchange disorders [1]. More specifically, ALI can lead to excessive or long-term activation of immune cells in the body, releasing destructive pro-inflammatory and pro-apoptotic mediators, disrupting the balance of inflammation/anti-inflammatory and oxidative/reductive processes, and causing cell apoptosis, leading to lung pathology [2]. In clinical practice, drugs such as dexamethasone and hydrocortisone are used to treat acute lung injury, but they can cause serious adverse reactions [3]. Therefore, exploring new drug-lead compounds from natural products to treat ALI remains crucial. Fruits are a vital dietary component, rich in diverse metabolites that contribute to health benefits. Studies on fruit metabolomics highlight the potential of natural fruit products in treating both acute and chronic diseases, offering promising insights for improving both nutritional value and therapeutic applications [4].

*Clausena lansium* (*C. lansium*) is a beautiful shrub or small tree with fruit that resembles grapes and citrus fruits, commonly known as Wampee, False, or Fool’s Curry [5]. *C. lansium* is native to southern China and widely planted in Fujian, Guangdong, Guangxi, south Guizhou, Hainan, Sichuan, southeastern Yunnan, and northern to central Vietnam [6]. In Taiwan and China, its leaves are used as folk medicine to treat respiratory diseases such as cough and asthma [7]. *L*-Clausenamide is a bioactive amide from *C. lansium* [6], which can alleviate the Aβ-induced oxidative stress in PC12 cells [8]. Moreover, the amide structure is important in many natural products and drugs, especially in potential drug screening for acute lung injury [9]. Therefore, *L*-Clausenamide is likely to be a critical active phytochemical in *C. lansium* that cures acute lung injury through improving the oxidative stress.

Network pharmacology is a novel technology that combines big data, artificial intelligence, and systems biology, and has been widely used for screening active compounds in natural products and studying their underlying molecular mechanisms [10,11]. More specifically, the SWISSADME database is used to predict the ADME parameters of the bioactive compounds [12]. The PharmMapper database can identify potential targets for bioactive compounds using a pharmacophore mapping approach [13]. The OMIM and GeneCards databases are commonly used databases for collecting disease targets [14,15]. The String and Metascape databases are used for the analysis of protein–protein interactions and KEGG&GO analysis [16,17]. Moreover, molecular simulation techniques and in vitro experiments can be used to further study the results of network pharmacology [18].

For the in vitro model, the A549 cells are lung carcinoma epithelial cells and have been commonly used for studying the therapeutic potential of natural products in acute lung injury [19]. Lipopolysaccharide, the outer membrane component of Gram-negative bacteria, is one cause of acute lung injury, which can lead to oxidative stress and apoptosis in A549 cells [20]. Shen et al. summarized that the LPS-induced acute lung injury model in a dose-dependent and time-dependent manner [19].

Based on this, this study combines network pharmacology, molecular simulation techniques, and in vitro validation to elucidate the effects and the potential mechanisms of *L*-Clausenamide on LPS-induced acute lung injury. Firstly, network pharmacology was used to identify the core targets and related pathways by which *L*-Clausenamide improves lipopolysaccharide (LPS)-induced acute lung injury through ROS. Subsequently, the interaction between *L*-Clausenamide and its central targets was studied through molecular docking and molecular dynamics. In addition, the in vitro LPS-induced acute lung injury model of A549 cells was further validated using *L*-Clausenamide. This study contributes to a deeper understanding of the therapeutic mechanism of *L*-Clausenamide in treating acute lung injury.

## 2. Materials and Methods

### 2.1. ADME Analysis and Target Prediction of L-Clausenamide

The structure information of *L*-Clausenamide was obtained from the PubChem database (https://pubchem.ncbi.nlm.nih.gov/ (accessed on 13 August 2024)). We performed the ADME analysis of *L*-Clausenamide through the SwissADME database (http://www.swissadme.ch/ (accessed on 27 February 2025)) [12]. We constructed the chemical structure by InDraw. PharmMapper (https://www.lilab-ecust.cn/pharmmapper/ (accessed on 13 August 2024)) was used to collect protein targets of *L*-Clausenamide (https://doi.org/10.1093/nar/gkx374). The running conditions were set for the Human Protein Targets Only. All collected protein targets were converted into gene names through the UniProt database (https://www.uniprot.org/ (accessed on 13 August 2024)) for further analysis [13].

### 2.2. The Collection of the Acute Lung Injury and ROS-Related Targets

The acute lung injury-related targets were collected from the Genecards (https://www.genecards.org/ (accessed on 13 August 2024)) and OMIM (https://www.omim.org/ (accessed on 13 August 2024)) databases using the keyword acute lung injury [14,15]. All collected relevant target information was processed uniformly through the UniProt database and was output as the gene names.

### 2.3. Identification of Overlapping Anti-Acute Lung Injury Targets of L-Clausenamide

Jvenn online tool (https://jvenn.toulouse.inra.fr/app/example.html (accessed on 13 August 2024)) was used to obtain interacting targets between the targets of *L*-Clausenamide, acute lung injury, and ROS [21]. The overlapping targets are the anti-acute lung injury targets of *L*-Clausenamide. The results were shown as a Venn diagram.

### 2.4. Protein–Protein Interaction (PPI) Analysis

The overlapping targets were imported into the String database (https://string-db.org/ (accessed on 13 August 2024)) for further analysis [16]. The model was set to multiple proteins, and the minimum required interaction score was set to medium confidence (0.400). Moreover, the organisms were set to Homo sapiens, and the analysis results were exported as a TSV file and subsequently imported into Cytoscape 3.9.1 for further analysis.

### 2.5. Network Construction of PPI Analysis and the Drug–Compound–Target–Disease Network

The PPI network was constructed by Cytoscape, and the original data was downloaded from the String as a TSV. file. The TSV. file was submitted to Cytoscape to generate the PPI network. Furthermore, Cytoscape was used to construct a network of *L*-Clausenamide, its anti-acute lung injury targets, and acute lung injury.

### 2.6. Screening the Core Targets and Central Targets of L-Clausenamide

The network of protein–protein interactions (PPI) was constructed and analyzed through Cytoscape software. The anti-acute lung injury targets with Betweenness, Closeness, and Degree centrality metrics exceeding the median were identified and collected. Similarly, the central targets were screened by the top 10 DC value, CC value, MCC value, and the MNC value.

### 2.7. GO and KEGG Enrichment Analysis

Metascape database (https://metascape.org/gp/index.html#/main/step1 (accessed on 28 August 2024)) was used to conduct gene ontology (GO) functional enrichment analysis and Kyoto Encyclopedia of Genes and Genomes (KEGG) pathway enrichment analysis to the core targets [17]. The items of GO enrichment analysis included cellular component (CC), biological process (BP), and molecular function (MF). The top 10 GO analysis items (BP, CC, and MF) and related KEGG pathway analysis results were displayed in the form of a dot plot. The dot plot was generated by the bioinformatics online platform SRplot (https://www.bioinformatics.com.cn (accessed on 28 August 2024)).

### 2.8. Molecular Docking

AutoDock Vina (v 1.5.7) and PyRx (v 0.8) software were used for molecular docking, following the method outlined by (https://doi.org/10.1016/j.foodhyd.2023.109467). The target proteins included serum albumin (ALB, 2BX8), RAC-alpha serine/threonine-protein kinase (AKT1, 3CQW), caspase-3 (CASP3, 1RHK), heat shock protein 90-alpha (HS90A, 3BMY), estrogen receptors (ESR1, 1NDE), epidermal growth factor receptor (EGFR, 1M17), matrix metalloproteinase-9 (MMP-9, 1GKD), and proto-oncogene tyrosine-protein kinase (SRC, 104F). The crystal structures of these proteins were sourced from the RCSB protein data bank (rcsb.org), while the three-dimensional structure of *L*-Clausenamide was obtained from the PubChem database (http://pubchem.ncbi.nlm.nih.gov (accessed on 13 August 2024)). Charges for *L*-Clausenamide were assigned using the CHARMM General Force Field (CGenFF) program. Prior to docking, water molecules and non-polar hydrogen atoms were removed from the protein structures, and polar hydrogens were added before applying Kollman united atom charges. The grid box was set to cover the entire protein structure, and the docking was performed with an exhaustiveness of 1000 runs. The resulting binding models were visualized using Discovery Studio 2019 (BIOVIA Corp., San Diego, CA, USA) and Visual Molecular Dynamics (VMD) software (v 1.9.3).

### 2.9. Materials

A 96-well, glass-bottom, black-edge plate for ATP (luminescence), ROS, and membrane depolarization (fluorescence) measurement, and a 35 mm confocal dish were purchased from Cellvis (Sunnyvale, CA, USA) for measurement by a multi-function microplate reader. The 35 mm, glass and clear bottom (15 mm), black-edge culture dish was purchased from Biosharp (Beijing, China) for bio-imaging under a super-resolution microscope. The DMEM/F12, 100× penicillin/streptomycin solution, and A549 cell line were purchased from Procell Life Science & Technology Co., Ltd. (Wuhan, China). The *L*-Clausenamide was purchased from TargetMol (Boston, MA, USA). Phosphate-buffered solution (PBS) and dimethyl sulfoxide (DMSO) solution were purchased from Beijing Solarbio Science & Technology Co., Ltd. (Beijing, China). Fetal bovine serum (FBS) was bought from Shanghai XP Biomed Ltd. (Shanghai, China). The LPS (ST1470), Enhanced cell counting kit-8 (CCK-8) reagent kit (C0041), CellTiter-LumiTM Plus II Cellular ATP level determination Kit, the mitochondrial membrane potential assay kit with JC-1 kits (C2006), Caspase-3 Activity, Mitochondrial Membrane Potential Detection Kit for Live Cell (C1073M), and the secondary anti-rabbit IgG (H+L) antibodies were purchased from Beyond Biotech Inc. (Shanghai, China). The DiBaC_4_ was purchased from GLPBio (Montclair, NJ, USA). Hochest 33342, Carboxy-H2DCFDA, and Mitotracker Deep Red were purchased from Invitrogen (Waltham, MA, USA). The HBmito Crimson was purchased from Genevivo Biotech (Nanjing, China). The CM7 sensor chips (Cat No 28957332) were purchased from GE Healthcare (Marlborough, MA, USA). The AKT1 protein (Cat No. HY-P74421) and AKT inhibitor (AKT-IN-1) (Cat No. HY-18296) were purchased from MCE (Monmouth Junction, NJ, USA). The Co-IP/WB Tissue/Cell Lysis Buffer (plus protease inhibitor), WB Fast Transfer Buffer, 5X SDS-PAGE Loading Buffer (Reduced), Robust ECL Solution, and Minute Block Blocking Buffer were purchased from Affinibody LifeScience Co., Ltd. (Wuhan, China). The primary antibodies against AKT, p-AKT, and cleaved caspase-3 were purchased from Affinity Biosciences (Cincinnati, OH, USA). The TBST Buffer Solution, 8% Fast-Cast Colorful (Green) Acrylamide Kit, and Rapid High-Resolution Running Buffer were purchased from Servicebio Technology Co., Ltd. (Wuhan, China).

### 2.10. Cell Culture and Treatment

A549 cells were seeded and cultured in DMEM/F12 (1:1) medium, 10% fetal bovine serum, and 1% 100× penicillin/streptomycin solution, at 37 °C, 5% CO_2_. Lipopolysaccharide (LPS) was used for modeling and set at 10 µg/mL according to the CCK8 cytotoxicity test.

After 24 h of culture in DMEM/F12 (1:1), A549 cells were treated with LPS-containing DMEM/F12 (1:1) for another 24 h of culture before other experiments. Concentration-gradient *L*-Clausenamide was applied with LPS together.

### 2.11. Cell Viability Determination

Commercial Cell Counting Kit-8 (CCK-8) was used for cell viability measurement in this study, following the user guidelines. Details of experimental procedures can be referred to in our previous study [18].

Concentration gradient *L*-Clausenamide (0, 11.5, 25, 50, 100, and 200 μM), LPS (0, 0.1, 1, 10, and 100 µg/mL) were introduced for 24 h treatment after A549 cells (~10,000 cells per well) seeding in a 96-well plate for 24 h.

### 2.12. ROS Measurement

Details of experimental procedures can be referred to in our previous study [22].

Carboxy-H2DCFDA was used in this study for intracellular ROS accumulation measurement at micro- (microscope) or macro-perspective (microplate reader).

A549 cells were cultured in a confocal dish (~900,000 cells per well) for super-resolution microscopy or a 96-well plate with black edge and glass bottom (~10,000 cells per well) and treated as above. Next, cells in confocal dishes were co-stained with Carboxy-H2DCFDA, following the user guidelines, and Hoechst 33342 at 5 μM at 37 °C, 5% CO_2_, lasting for 30 min. Afterwards, images were visualized via super-resolution fluorescent microscope. For fluorescent determination at bulk culture level, cells were seeded in 96-well plate with a black edge and glass bottom, and treated and dyed the same as above. Relative Fluorescent Unit (RFU) was measured by a microplate reader and normalized by dividing by cell number.

### 2.13. Membrane Potential Measurement

Details of experimental procedures can be referred to in our previous study [22].

DiBAC4 was used in this study for membrane potential measurement.

A549 cells were cultured in a confocal dish (~900,000 cells per well) for super-resolution microscopy or 96-well plate with black edge and glass bottom (~10,000 cells per well) and treated as above. Next, cells in confocal dishes were co-stained with DiBAC4, following the user guidelines, and Hoechst 33342 at 5 μM at 37 °C, 5% CO_2_, lasting for 30 min. Afterwards, images were visualized via super-resolution fluorescent microscope. For fluorescent determination at bulk culture level, cells were seeded in 96-well plate with a black edge and glass bottom, and treated and dyed the same as above. RFU was measured by a microplate reader (Molecular Devices, Sunnyvale, CA, USA) and normalized by dividing by cell number.

### 2.14. Mitochondrial Membrane Potential Measurement

Details of experimental procedures can be referred to in our previous study [22].

JC-1 was used in this study for membrane potential measurement.

A549 cells were cultured in a confocal dish (~900,000 cells per well) for super-resolution microscopy or 96-well plate with black edge and glass bottom (~10,000 cells per well) and treated as above. Next, cells in confocal dishes were co-stained with JC-1, following the user guidelines, and Hoechst 33342 at 5 μM at 37 °C, 5% CO_2_, lasting for 30 min. Afterwards, images were visualized via super-resolution fluorescent microscope (ZEISS, Oberkochen, Germany). For fluorescent determination at bulk culture level, cells were seeded in 96-well plate with a black edge and glass bottom, and treated and dyed the same as above. RFU was measured by a microplate reader and normalized by dividing by cell number.

### 2.15. Intracellular ATP Determination

Details of experimental procedures can be referred to in our previous study [22].

Intracellular ATP amount was determined by a commercial kit according to the manufacturer’s instructions. A549 cells (~10,000 cells per well) were seeded in 96-well plate with black edge and glass bottom and treated the same as above. The relative luminescent unit (RLU) was measured by a microplate reader and normalized by dividing by cell number.

### 2.16. Apoptotic Cell Measurement and Mitochondrial Morphology Observation

Details of experimental procedures can be found in references [23,24,25]. FITC-DEVDG-peptide, TUNEL assay, and Annexin-V conjugated with mCherry were used for apoptotic cell level measurement at either single-cell or bulk culture level. Mitotracker Deep Red or HBmito Deep Red was used for sub-cellular level observation of mitochondrial morphology.

A549 cells (~900,000 cells per well) were cultured in a confocal dish and treated the same as above. Afterwards, cells were washed thrice by PBS to wash the medium out. Subsequently, live cells were co-stained by Intracellular cleaved caspase-3 activity measurement probe (FITC-DEVDG-peptide), or Mitochondrial morphology specific probe (Mitotracker Deep Red or HBmito Deep Red) or TUNEL assay, following the user guide provided by the kit, and Hoechst 33342 at 5 μM at 37 °C, 5% CO_2_, lasting for 30 min. Next, cells were washed thrice before imaging. Afterwards, images were visualized via super-resolution fluorescent microscope. For fluorescent determination at bulk culture level, cells (~10,000 cells per well) were seeded in 96-well plate with black edge and glass bottom, and treated and dyed the same as above. RFU was measured by a microplate reader and normalized by dividing by cell number.

### 2.17. Microscope and Image Analysis

Details of experimental procedures can be referred to in our previous study [22].

The observations of intracellular ROS (Carboxy-H2DCFDA), Mitochondrial membrane depolarization (JC-1) and morphology (Mitotracker Deep Red or HBmito Deep Red), apoptotic cells (FITC-DEVDG-peptide, Annexin V-mCherry or TUNEL), and chromosome status (Hoechst 33342) determination were under Elyra 7 with Lattice SIM2 super-resolution fluorescent microscope (ZEISS, Oberkochen, Germany) containing 63× oil immersion objective. During image acquisition, cells were in a humidified chamber maintained at 37 °C in the presence of 5% CO_2_.

Original images captured by super resolution microscope (ZEISS, Oberkochen, Germany), were reconstructed by ZEISS ZEN software black edition (Version 3.2) following the standard protocol before further processed and exported by ZEISS ZEN software blue edition (Version 3.6). All original figures before reconstruction are all provided in the Supplementary Materials (Figures S1–S8).

For cellular fluorescence measurement, image processing and analysis was conducted through Fiji (ImageJ, version 2.14.0).

### 2.18. SPR Analysis

The CM7 sensor chip and Biacore 8K+ system were applied in this study for Surface plasmon resonance (SPR) experiments according to the user guidelines and reference [26]. *L*-Clausenamide (Targetbio; Cat No. T32614) and the AKT1 protein (MCE; Cat No. HY-P74421) were prepared according to the instruction manual.

### 2.19. Western Blot Analysis

A549 cells were cultured in a 6-well plate (~900,000 cells per well) and treated the same as above. The Western blot was conducted after cells were harvested, and the details of the experimental procedure are referred to in our previous study [22].

### 2.20. Statistical Analysis

All tests for this study were conducted at least thrice. One-way analysis of variance (ANOVA) is used for statistical analysis, and the data is represented as mean ± SD. Tukey’s test is used for analysis on GraphPad Prism 9.5 software, with statistical significance set at *p* < 0.05.

## 3. Results

### 3.1. The ADME Analysis of L-Clausenamide

ADME (Absorption, Distribution, Metabolism, and Excretion) analysis through machine learning models is an efficient drug discovery and development strategy. Moreover, Lipinski rules are used to assist in screening compounds with good pharmacokinetic properties. Meeting this rule means that the drug has good bioavailability and oral absorption, and has further research value [27]. The study used the SwissADME database for ADME analysis of *L*-Clausenamide and traced the most critical results with reference ranges (Table 1). The results indicate that *L*-Clausenamide can meet Lipinski rules, which means it has good pharmacokinetic properties. In addition, *L*-Clausenamide can be absorbed through the blood–brain barrier and has a good gastrointestinal absorption and bioavailability score, which means that *L*-Clausenamide has good bioavailability and great potential to develop as a medicine.

### 3.2. The Anti-Acute Lung Injury Targets of L-Clausenamide

**Figure 1:**
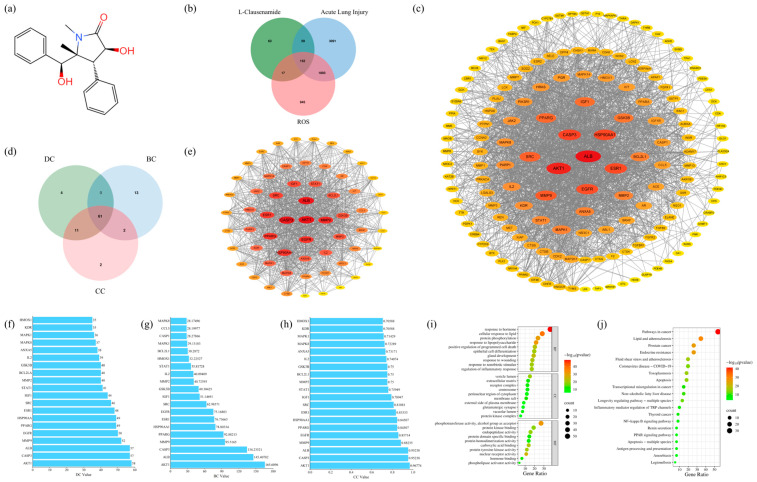
The anti-acute lung injury targets of *L*-Clausenamide: (**a**) The structure of *L*-Clausenamide drawn by ChemDraw. (**b**) The Venn diagram between the targets of *L*-Clausenamide, acute lung injury, and ROS. (**c**) The PPI network of the 152 anti-acute lung injury targets. (**d**) The Venn diagram between the targets with the DC, BC, and CC topological values higher than the median. (**e**) The PPI network of 61 core anti-acute lung injury targets. (**f**) The anti-acute lung injury targets with the top 20 DC value. (**g**) The anti-acute lung injury targets with the top 20 BC value. (**h**) The anti-acute lung injury targets with the top 20 CC value. (**i**) The dot plot of the KEGG enrichment result. (**j**) The dot plot of the GO enrichment analysis. BP, Biological Process; CC, Cellular Components; MF, Molecular Function.

The structure of *L*-Clausenamide was constructed on ChemDraw 22.0.0 based on the information from PubChem, as shown in Figure 1a. Moreover, this structure was used to collect a total of 296 protein targets of *L*-Clausenamide on the PharmMapper database. The protein targets of acute lung injury were obtained through the DisGeNet database, with 4926 protein targets, and the OMIM database, with 381 protein targets. After removing duplicates, a total of 5195 acute lung injury protein targets were obtained. The ROS-related protein targets were obtained through the DisGeNet database, and a total of 3007 were protein targets. Taken together (Figure 1b), *L*-Clausenamide has 152 targets for attenuating acute lung injury by improving reactive oxygen species (ROS).

### 3.3. Protein–Protein Interaction (PPI) Analysis and Core Target Screening

The protein–protein interaction (PPI) analysis was conducted through the String database based on the 153 anti-acute lung injury targets. And we have reconstructed it through Cytoscape for the visualization in Figure 1c. The PPI network consists of 151 nodes and 1928 edges, indicating the complexity of the network. Based on Figure 1c, the degree (DC), Betweenness (BC), and Closeness (CC) were calculated through Cytoscape, and the core targets were screened through the median of DC, BC, and CC (Figure 1d). The deeper the color of the protein node, the larger the node, and the more central the position in Figure 1e, indicating the higher the DC value of the protein.

The screening of the core target can make research become more precise and scientific [18]. Moreover, the 61 anti-acute lung injury core targets of *L*-Clausenamide were obtained using Venn diagrams to identify targets with DC, CC, and BC values higher than the median. Similarly, these 61 anti-acute lung injury core targets were also utilized to conduct the PPI analysis and reconstructed through Cytoscape (Figure 1e). The top 20 core targets are shown in Figure 1f–h. AKT1 is the top 1 in these three topological values, which is the most important protein target of *L*-Clausenamide improving acute lung injury. The topological values of these proteins represent their importance in this PPI network [28].

### 3.4. Result of GO and KEGG Enrichment Analysis

The Metascape database was used to conduct a GO enrichment analysis of 61 core anti-acute lung injury targets [17]. A total of 1587 BP, 79 CC, and 164 MF terms were enriched and met *p* < 0.01. The top 10 enrichment terms of BP, CC, and MF were presented in Figure 1g. The GO enrichment analysis results indicate that the gene target involves multiple BPs, including response to hormone, cellular response to lipid, protein phosphorylation, response to lipopolysaccharide, positive regulation of programmed cell death, etc. In the enriched CC category, the core target involves the vesicle lumen, extracellular matrix, receptor complex, centrosome, perinuclear region of cytoplasm, etc. Moreover, based on the GO enrichment result, MF ontologies include phosphotransferase activity, alcohol group as acceptor, protein kinase binding, endopeptidase activity, protein domain-specific binding, etc.

The 61 core targets obtained from Section 2.2 were imported into the Metascape database for KEGG Pathway enrichment analysis [17]. The result is shown as a dot plot in Figure 1h. The results of KEGG enrichment analysis showed that the molecular mechanism of *L*-Clausenamide in treating acute lung injury through ROS may involve pathways in cancer, lipid and atherosclerosis, prostate cancer, endocrine resistance, fluid shear stress and atherosclerosis, coronavirus disease COVID-19, toxoplasmosis, and apoptosis. Especially apoptosis might be caused by ROS and downstream of the ROS. *L*-Clausenamide may alleviate acute lung injury through apoptosis and ROS. In this case, further in vitro experiments were focused on ROS and apoptosis.

### 3.5. Central Target Screening

**Figure 2:**
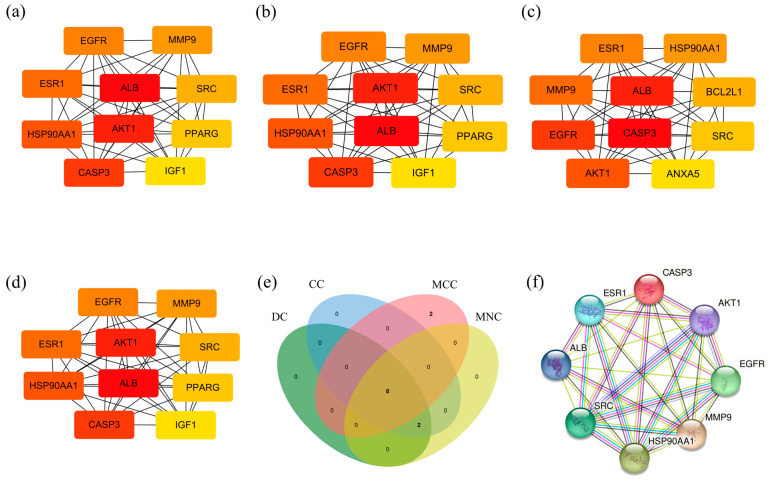
The central targets screening for molecular docking and molecular dynamics: (**a**) The anti-acute lung injury targets with top 10 DC value. (**b**) The anti-acute lung injury targets with top 10 CC value. (**c**) The anti-acute lung injury targets with top 10 MCC value. (**d**) The anti-acute lung injury targets with top 10 MNC value. (**e**) The Venn diagram between the targets with top 10 DC value, CC value, MCC value, and MNC value. (**f**) The PPI analysis of the central targets.

We screened the Central targets of *L*-Clausenamide, which are the most promising targets in treating acute lung injury through regulating ROS. After analyzing the PPI analysis of 152 anti-acute lung injury targets through the String database, the results were imported into Cytoscape for network analysis using cytoHubba. Moreover, the targets of the PPI network with top 10 Degree (Figure 2a), Closeness (Figure 2b), MCC (Figure 2c), and MNC (Figure 2d) values were shown, respectively. The Venn diagram was used to collect the interacting targets of the above four networks as central targets, including CASP3, AKT1, EGFR, MMP9, HSP90AA1, SRC, ALB, and ESR1. Based on Figure 1f, AKT1 is the most important protein target of *L*-Clausenamide, alleviating acute lung injury, which is also one of the central targets. Another one of the central targets, the CASP3 protein, can regulate apoptosis after being cleaved and activated, and is regulated by AKT1. Taken together, the *L*-Clausenamide may inhibit the cleavage of caspase-3 and apoptosis through targeting AKT1.

### 3.6. Result of Molecular Docking

**Figure 3:**
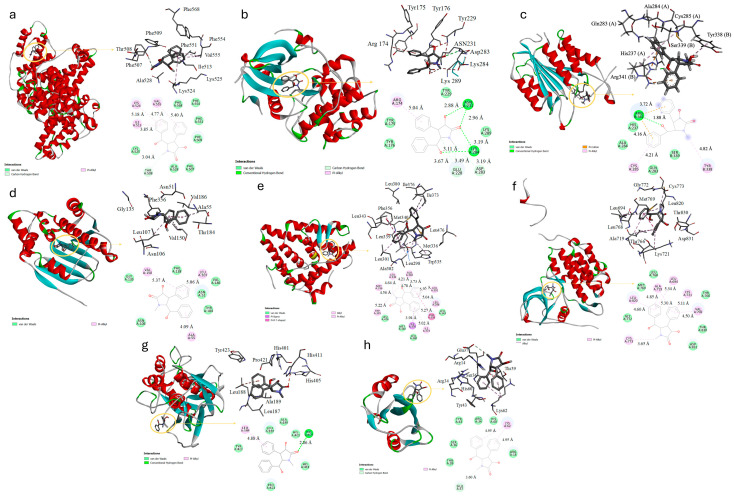
The 3D zoom-out and close-up images, along with the 2D molecular interaction images between *L*-Clausenamide and (**a**) serum albumin (ALB); (**b**) RAC-alpha serine/threonine-protein kinase (AKT1); (**c**) caspase-3 (CASP3); (**d**) heat shock protein 90-alpha (HS90A); (**e**) estrogen receptors (ESR1); (**f**) epidermal growth factor receptor (EGFR); (**g**) matrix metalloproteinase-9 (MMP-9); (**h**) proto-oncogene tyrosine-protein kinase (SRC).

After the screening of central targets, molecular docking was performed between the central targets and *L*-Clausenamide. The 3D zoom-out and close-up images, along with the 2D molecular interaction images between *L*-Clausenamide and various central targets, are presented in Figure 3a–h. Notably, the 3D zoom-out image in Figure 3a exclusively displays the interacting chain A of the ALB. The docking results indicated that ESR1 had the lowest binding score of −7.4 kcal/mol, reflecting the strongest binding affinity with *L*-Clausenamide. This was followed by MMP-9 (−7.2 kcal/mol), HSP90AA1 (−7.1 kcal/mol), and AKT1 (−6.9 kcal/mol). ALB, CASP3, and EGFR all shared the same binding affinity of −6.8 kcal/mol, while SRC displays the weakest binding affinity to *L*-Clausenamide with a score of −5.4 kcal/mol. AKT1 also showed a good binding affinity with *L*-Clausenamide, and a further binding validation experiment should be performed through the SPR analysis.

More specifically, Figure 3e illustrates that six pi–alkyl hydrophobic interactions, three alkyl interactions, one pi–sigma interaction, one pi–pi T-shaped interaction, and four van der Waals interactions were established between *L*-Clausenamide and ESR1. In contrast, *L*-Clausenamide and SRC formed two pi–alkyl interactions, one carbon–hydrogen bond, and seven van der Waals interactions. The *L*-Clausenamide–MMP-9 interaction features one pi–alkyl bond, one hydrogen bond, and five van der Waals forces, while *L*-Clausenamide and HS90A are connected by three pi–alkyl bonds and five van der Waals forces. For AKT1, the interactions include one pi–alkyl bond, four hydrogen bonds, two carbon–hydrogen bonds, and four van der Waals forces. Even though ALB, CASP3, and EGFR share the same binding affinity score, the types of interacting bonds varied. Specifically, CASP3 interacted with *L*-Clausenamide through two pi–cation interactions, including one between a phenyl ring of *L*-Clausenamide and the amino acid residue Arg341 at a distance of 4.16 Å, another between the same amino acid residue but the other phenyl ring at 3.72 Å on protein chain B. Additionally, two pi–alkyl hydrophobic bonds were formed between a phenyl ring on *L*-Clausenamide and Cys285 on protein chain A at 4.21 Å, and the other one is between Tyr338 on protein chain B and a carbon atom of *L*-Clausenamide A hydrogen bond was also established between Arg341 on protein chain B and the hydroxyl group of *L*-Clausenamide at a distance of 1.88 Å, along with four van der Waals interactions. In comparison, the interaction between ALB and *L*-Clausenamide involved four pi–alkyl bonds, one carbon–hydrogen bond, and seven van der Waals forces, while EGFR and *L*-Clausenamide interacted through six pi–alkyl bonds, one alkyl bond, and six van der Waals forces.

### 3.7. L-Clausenamide Inhibits LPS-Facilitated Cell Viability Decrease

**Figure 4:**
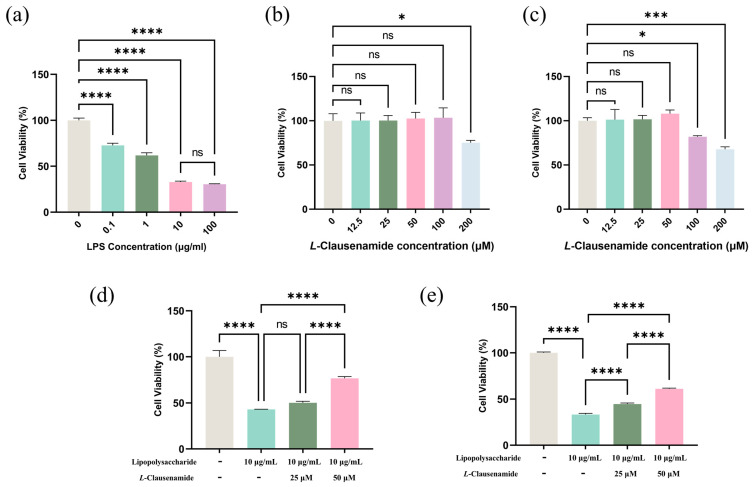
*L*-Clausenamide alleviates LPS-treated A549 cell viability decrease: (**a**) The effect of LPS on the cell viability of A549 cells after treating for 24 h. (**b**) The effect of *L*-Clausenamide on the cell viability of A549 cells after treating for 24 h. (**c**) The effect of *L*-Clausenamide on the cell viability of A549 cells after treating for 48 h. (**d**) The alleviation effect of *L*-Clausenamide (25 μM or 50 μM) on LPS (10 µg/mL)-facilitated A549 cell viability loss after treating for 24 h. (**e**) The alleviation effect of *L*-Clausenamide (25 μM or 50 μM) on LPS (10 µg/mL)-facilitated A549 cell viability loss after treating for 48 h. All results are presented as mean ± S.D., and the experiments were repeated in triplicate. ns, *p* > 0.05; *, *p* < 0.05; ***, *p* < 0.001; ****, *p* < 0.0001.

Firstly, we applied the CCK8 method to evaluate the cell viability of A549 cells after LPS introduction with various concentrations (0, 0.1, 1, 10, 100 μg/mL) for 24 h. Exposure to 10 µg/mL LPS will cause over 50% cell viability loss, while a 10-fold enhancement do not cause a significant change in cell viability between 10 and 100 µg/mL. It implied 10 µg/mL was suitable for modeling (Figure 4a). Next, we measured the cytotoxicity of *L*-Clausenamide on A549 cells for 24 or 48 h treatment (Figure 4b,c). Although at 24 h, 100 μM *L*-Clausenamide did not show a significant inhibitory effect, treatment extended to 48 h discovered that it caused a significant change in cell viability. Accordingly, it implied that 25 and 50 µM were set for a treatment concentration limit without cytotoxicity. Afterwards, we compared the effects of 25 and 50 µM *L*-Clausenamide on cell viability loss prevention on LPS-induced A549 cells (Figure 4d,e). For 48 h treatment, both 25 and 50 µM *L*-Clausenamide significantly alleviate cell viability loss, while at the 24 h timepoint, only 50 µM *L*-Clausenamide obviously prevents cell viability loss. To well present the effectiveness of *L*-Clausenamide and ensure enough survival cells for observation under a super-resolution microscope after modeling, we set the modeling condition at 24 h inducement by 10 µg/mL LPS and treatment conditions at 25 and 50 µM *L*-Clausenamide for 24 h [20,29].

### 3.8. L-Clausenamide Inhibits LPS-Facilitated Intracellular ROS Accumulation

**Figure 5:**
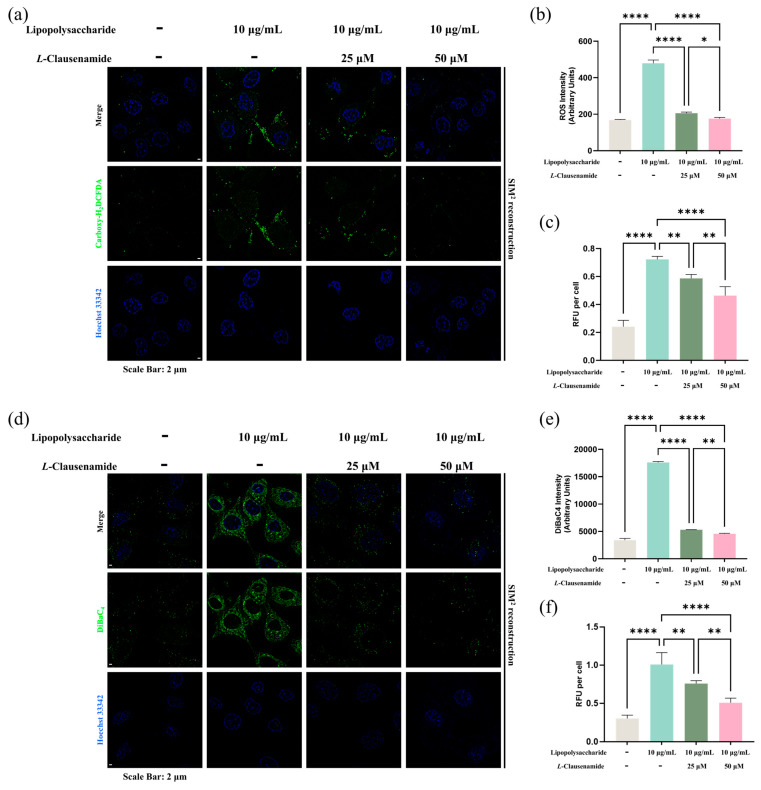
*L*-Clausenamide alleviates LPS-facilitated intracellular ROS accumulation and membrane potential decrease: (**a**) Fluorescent images of A549 cells co-staining with Hoechst 33342 (blue) and Carboxy-H_2_DCFDA (green). Scale bars, 2 μm. (**b**) Semi-quantitative analysis based on original images of ROS intensity. (**c**) Normalized Relative fluorescent unit (RFU) representing ROS accumulation, which is divided for cell numeration. (**d**) Fluorescent images of A549 cells co-stained with Hoechst 33342 (blue) and DiBaC_4_ (green). Scale bars, 2 μm. (**e**) Semi-quantitative analysis based on original images of fluorescence signal of DiBaC4 intensity by ImageJ. (**f**) Normalized relative fluorescent unit (RFU) representing membrane potential change (DiBaC4), which is divided for cell numeration. All results are presented as mean ± S.D., and the experiments were repeated in triplicate. *, *p* < 0.05; **, *p* < 0.01; ****, *p* < 0.0001.

To study how *L*-Clausenamide rescues the cell viability decrease induced by LPS, we started from intracellular ROS generation, and used the Carboxy-H2DCFDA, with Hoechst 33432, staining at a single-cell level analysis (Figure 5a). In normal cells without LPS stimuli, few green foci (ROS signal) were observed in the cytoplasm or nucleus region. Meanwhile, in LPS-induced cells, numerous bright green fluorescent foci distributions were clearly shown in either the cytoplasm or nucleus, indicating ROS accumulation might stress DNA and intracellular organelles, facilitating a cell viability decrease. *L*-Clausenamide introduction alleviates the formation of green foci accumulation in a dose-dependent manner (Figure 5a). A semi-quantitative analysis representing fluorescence observed under a microscope was conducted, with the results indicating that intracellular ROS accumulation is significantly enhanced in the LPS-inducement group compared to the normal control, and the introduction of 25 μM and 50 μM *L*-Clausenamide can decrease the ROS level significantly at the single-cell level (Figure 5b). Meanwhile, we also studied the ROS accumulation level at bulk culture (96-well plate) via the same dye (Figure 5c). The result is also similar and matches the above one, the semi-quantitative analysis. Both results are also similar to the cell viability measurement result above. Intracellular ROS accumulation can damage the cellular and mitochondrial membranes, facilitating the loss of potential and depolarization, which is critical and important for ATP synthesis and energy supply [30]. Collectively, consequences imply classic ROS-mediated caspase-3-dependent mitochondrial apoptosis as the clue for study, which might be crucial for *L*-Clausenamide-facilitated cell viability loss prevention in the LPS-stressed cellular model.

### 3.9. L-Clausenamide Inhibits LPS-Facilitated Mitochondrial Membrane Potential Loss and ATP Decrease

Since a previous study showed intracellular ROS accumulation facilitates classical mitochondrial apoptosis phenotypes like membrane potential loss and ATP decrease, we aim to further study whether *L*-Clausenamide facilitated LPS-induced ROS alleviation contributes to mitochondrial recovery.

Firstly, we used DiBaC_4_, a general indicator of membrane potential, and Hoechst 3342 co-staining to study changes in total membrane potential (Figure 5d). Our result showed that the DiBAC_4_ fluorescence in the LPS-induced group increases (green), indicating LPS drives cellular membrane integrity and potential abnormality. But treatment with either 25 or 50 μM *L*-Clausenamide can alleviate the green fluorescence accumulation, indicating alleviation of total membrane potential loss and depolarization. Moreover, a semi-quantitative analysis based on fluorescent level was conducted, and the results showed that the total cellular membrane potential decreased by LPS could be alleviated by concentration-dependent treatment of *L*-Clausenamide at the single-cell level (Figure 5e). Also, the fluorescent signal at the built culture level of DiBAC_4_ (RFU) was measured, and the result is also similar to the observations under a super-resolution microscope (Figure 5f). This result implies that mitochondrial membrane potential depolarization might be attributed to LPS stimuli, which could be alleviated by *L*-Clausenamide introduction.

**Figure 6:**
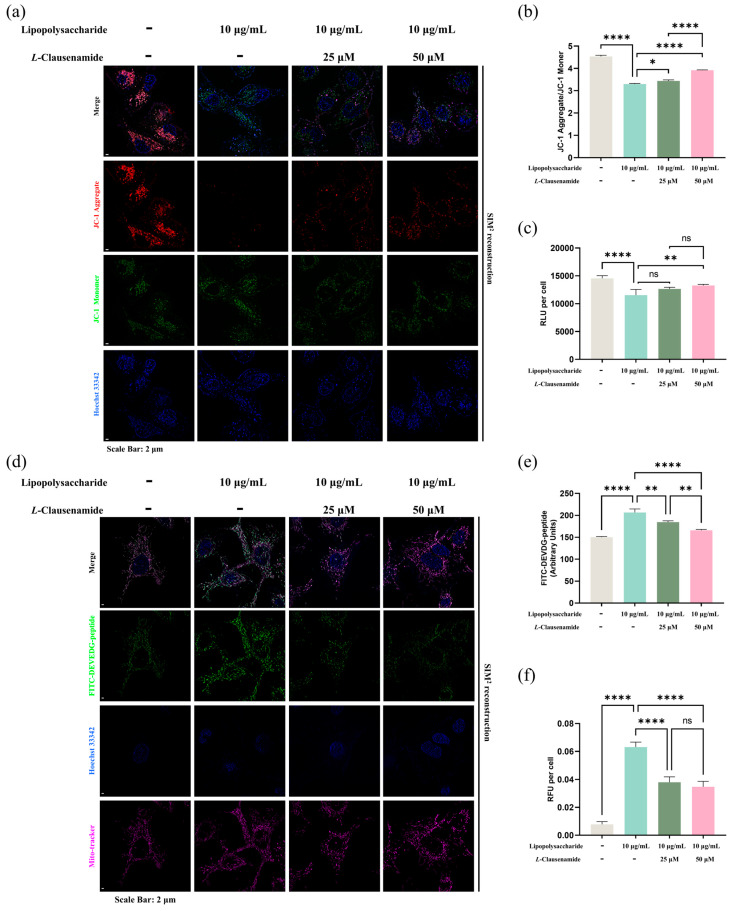
*L*-Clausenamide alleviates LPS-facilitated mitochondrial membrane potential, ATP level decrease, caspase-3 cleavage activity, and mitochondrial morphological change: (**a**) Fluorescent images of A549 cells co-staining with Hoechst 33342 (blue) and JC-1 (green or red). Scale bars, 2 μm. (**b**) Semi-quantitative analysis based on original images of fluorescence signal of JC-1 intensity. (**c**) Normalized relative luminescent unit (RLU) representing ATP level, which is divided by cell numeration. (**d**) Fluorescent images of A549 cells co-staining with Hoechst 33342 (blue), FITC-conjugated DEVDG-based peptide (green), and mito-tracker deep red. Scale bars, 2 μm. (**e**) Semi-quantitatively analysis based on original images of fluorescence signal of cleaved FITC-conjugated DEVDG-based peptide (green) intensity by ImageJ. (**f**) Normalized relative luminescent unit (RLU) representing cleaved FITC-conjugated DEVDG-based peptide (green, which is divided for cell numeration. All results are presented as mean ± S.D., and the experiments were repeated in triplicate. ns, *p* > 0.05; *, *p* < 0.05; **, *p* < 0.01; **** *p* < 0.0001.

Since DiBAC4 indicates the membrane potential generally, without organelle discrimination [31], we next applied the mitochondrial-specific membrane potential dye, JC-1, with Hoechst 33342, for direct observation of the mitochondrial membrane depolarization level (Figure 6a). As the most-used and well-tested mito-membrane-specific dye for membrane potential determination, it mainly distributes in the mitochondrial matrix for proton motive force (PMF) and forms an aggregate in normal cells, which shows a strong red fluorescent signal (570~620 em) when excited with a 561 nm laser. However, during apoptosis or other stressful conditions, loss of mitochondrial membrane potential facilitates JC-1 aggregation dispersion to the monomer, which shows a strong green fluorescent signal (420~480 em) when excited with a 488 nm laser. Accordingly, the ratio of red and green fluorescence and the location of fluorescence reflect the mitochondrial membrane potential status [32]. After SIM^2^ reconstruction, it is clearly shown that green and red fluorescence are co-located, which implies the membrane potential level of the mitochondria. However, attributed to the LPS stimulation, enhancement of green fluorescence alongside a red fluorescent signal decrease is clearly seen, indicating a mitochondrial membrane potential decrease. However, this phenotype can be rescued due to *L*-Clausenamide introduction (Figure 6a). Meanwhile, the semi-quantification analysis supports our results, showing that depolarization alleviation was due to *L*-Clausenamide, presented via JC-1 aggregate (red) to JC-1 monomer (green) (Figure 6b).

Moreover, ATP is the energy currency produced by the mitochondria via energetic metabolism, regarded as an important hallmark of mitochondrial normality. So, the ATP amount was also measured and presented via RLU (Figure 6c). This result indicated that the LPS stimuli decrease intracellular ATP levels in the bulk culture (96-well plate) and disrupt the mitochondrial energy metabolism. However, the introduction of *L*-Clausenamide, in a concentration-dependent manner, alleviates the ATP decrease.

To summarize, we used two completely different methods and fluorescent dyes and found *L*-Clausenamide with rescue capability in mitochondrial depolarization and function loss.

### 3.10. L-Clausenamide Inhibits LPS-Facilitated Mitochondrial Morphological Abnormality, Caspase-3 Activity, and DNA Fragmentation

Since *L*-Clausenamide alleviates LPS-induced ROS accumulation, ATP decreases, and the cellular and mitochondrial membrane potential decreases, all of which are classic mitochondrial apoptotic phenotypes; accordingly, we tried to further study whether *L*-Clausenamide treatment alleviates mitochondrial morphological abnormality and cleaved caspase-3 activity. We used a FITC-conjugated DEVDG-based peptide probe (apoptotic detection probe), Mitotracker Deep Red (mitochondrial-specific probe for morphology), and Hoechst 33342 co-staining to observe apoptosis and mitochondrial integrity. Meanwhile, for the apoptotic probe, FITC is conjugated with the negatively charged fulfilled peptide containing the DEVDG sequence, which can be recognized and digested by the activated and cleaved caspase-3. Only if the DEVDG linker sequence is successfully digested could strong green fluorescence representing apoptosis and cleaved caspase-3 activity be detected. LPS stimuli induce a strong green fluorescent signal formation, representing strong cleaved caspase-3 activity and apoptotic level alongside mitochondrial morphological changes in abnormality like orbicular, shortened, and inflated. However, due to *L*-Clausenamide treatment, green fluorescence was decreased alongside mitochondrial distortion alleviation (Figure 6d).

The semi-quantitative analysis at the single-cell level was conducted, and the result discovered that *L*-Clausenamide treatment facilitates intracellular activated caspase-3 cleavage activity that significantly decreases in comparison with the LPS-inducement group at the single-cell level (Figure 6e). Furthermore, we also conducted research on caspase-3 cleavage activity in a bulk culture via a microplate reader and the same dye (FITC-conjugated DEVDG peptide) (Figure 6f). The results of this semi-quantitative analysis are also similar and cross-verify the result presented by RFUs.

**Figure 7:**
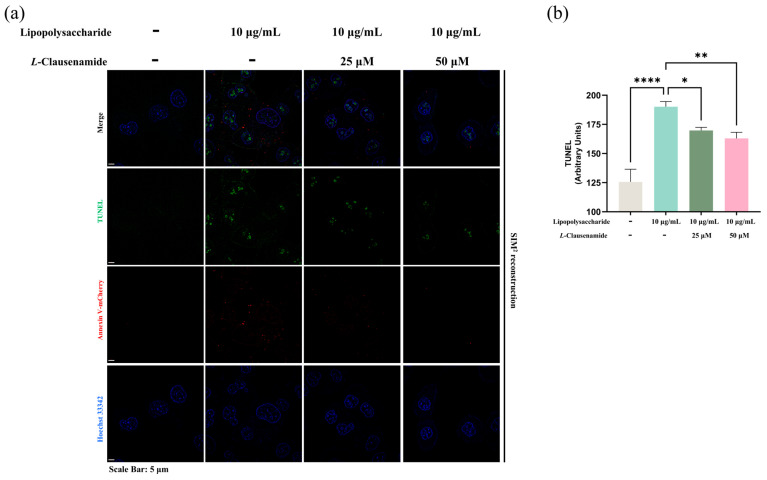
*L*-Clausenamide alleviates LPS-facilitated apoptosis: (**a**) Fluorescent images of A549 cells co-staining with Hoechst 33342 (blue), TUNEL (green), and Annexin V-mCherry (Red). Scale bars, 5 μm. (**b**) Semi-quantitative analysis based on original images of fluorescence signal of TUNEL intensity. All results are presented as mean ± S.D., and the experiments were repeated in triplicate. *, *p* < 0.05; **, *p* < 0.01; **** *p* < 0.0001.

Besides the activation of cleaved caspase-3, and mitochondrial membrane depolarization and morphological change, all of which are classical characteristics of apoptosis, we also applied the TUNEL assay, reflecting the DNA damage level and co-staining with Annexin V-conjugated with mCherry, a bio-marker of early apoptotic cells, for cross-verification of the apoptotic level alleviation of LPS-treated A549 cells by *L*-Clausenamide. LPS facilitates strong green fluorescent aggregation (TUNEL), which is co-located with blue fluorescence (Hoechst), alongside the formation of red fluorescence (Annexin-V mCherry) (Figure 7a). It clearly indicates that LPS causes DNA fragmentation in early apoptotic A549 cells. However, *L*-Clausenamide treatment alleviates green fluorescent aggregation alongside red fluorescence, implying its effect on DNA damage and apoptosis alleviation. Also, the semi-quantitative analysis of the TUNEL assay supported this finding (Figure 7b).

To summarize, we confirmed the apoptotic alleviation effect of *L*-Clausenamide on LPS-induced A549 cells via applying the multiple apoptosis detection method.

### 3.11. Molecular Mechanism of L-Clausenamide Improving the LPS-Induced Acute Lung Injury

**Figure 8:**
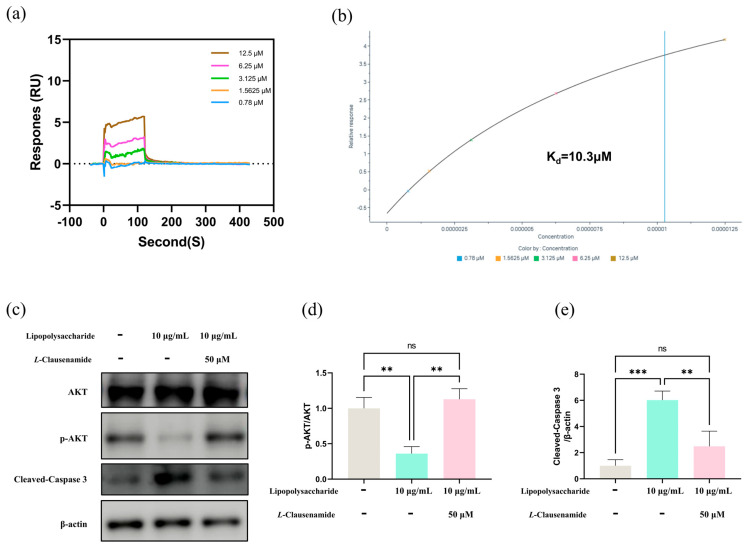
The molecular mechanism of *L*-Clausenamide: (**a**,**b**) The SPR analysis between AKT1 and *L*-Clausenamide. (**c**) The Western blot analysis of AKT, p-AKT, Cleaved Caspase-3, and β-actin. (**d**) The relative protein level of p-AKT/AKT. (**e**) The relative protein level of Cleaved Caspase-3/β-actin. All results are presented as mean ± S.D., and the experiments were repeated in triplicate. ns, *p* > 0.05; **, *p* < 0.01; ***, *p* < 0.001.

To further study the molecular mechanism of *L*-Clausenamide in improving acute lung injury, an SPR analysis and Western blot analysis were performed to validate that *L*-Clausenamide inhibits the cleavage of caspase-3 and apoptosis through targeting AKT1 (Figure 8a). The result clarified the direct interaction of *L*-Clausenamide with AKT1. *L*-Clausenamide did bind to AKT1 in a concentration-dependent manner with a Kd value of 10.3 μM (Figure 8b).

After studying the binding affinity between *L*-Clausenamide and AKT1, we further conducted a Western blot experiment to discuss the relationship between phosphorylation of the AKT amount and the activated, cleaved caspase-3 protein level (Figure 8c). The result showed that the *L*-Clausenamide can alleviate the decrease in the phosphorylation of AKT induced by the LPS (Figure 8d). Moreover, LPS-induced cleavage of caspase-3 was also inhibited by *L*-Clausenamide (Figure 8e). Taken together, *L*-Clausenamide can target AKT1 and increase its phosphorylation to inhibit the cleavage of caspase-3.

### 3.12. AKT Inhibitor Reduces the Apoptotic Alleviation Effect of L-Clausenamide on LPS-Induced A549 Cells

**Figure 9:**
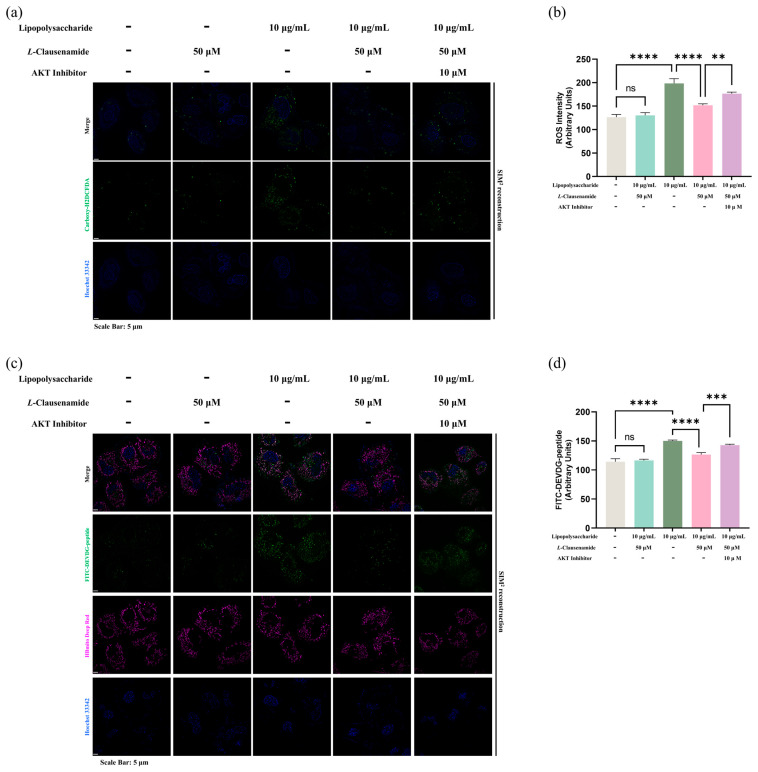
AKT inhibitors affect the improving effect of *L*-Clausenamide in LPS-facilitated ROS generation, caspase-3 cleavage activity, and mitochondrial morphological change: (**a**) Fluorescent images of A549 cells co-staining with Hoechst 33342 (blue) and Carboxy-H_2_DCFDA (green). Scale bars, 5 μm. (**b**) Semi-quantitatively analysis based on original images of ROS intensity by ImageJ. (**c**) Fluorescent images of A549 cells co-staining with Hoechst 33342 (blue), FITC-conjugated DEVDG-based peptide (green), and HBmito deep red. Scale bars, 5 μm. (**d**) Semi-quantitatively analysis based on original images of fluorescence signal of cleaved FITC-conjugated DEVDG-based peptide (green) intensity by ImageJ. All results are presented as mean ± S.D., and the experiments were repeated in triplicate. ns, *p* > 0.05; **, *p* < 0.01; ***, *p* < 0.001; **** *p* < 0.0001.

**Figure 10:**
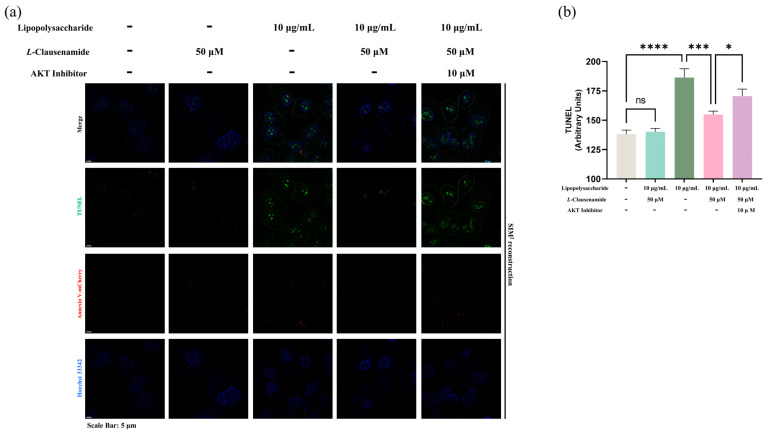
AKT inhibitor affects the improving effect of *L*-Clausenamide in LPS-facilitated apoptosis: (**a**) Fluorescent images of A549 cells co-staining with Hoechst 33342 (blue), TUNEL (green), and Annexin V-mCherry (Red). Scale bars, 5 μm. (**b**) Semi-quantitative analysis based on original images of fluorescence signal of TUNEL intensity by ImageJ. All results are presented as mean ± S.D., and the experiments were repeated in triplicate. ns, *p* > 0.05; *, *p* < 0.05; ***, *p* < 0.001; **** *p* < 0.0001.

Since we have confirmed that *L*-Clausenamide can bind to AKT, then increase the phosphorylation of AKT and inhibit the cleaved caspase-3, we next aim to directly link that increased AKT phosphorylation mediated by *L*-Clausenamide is critical in its role in apoptosis amelioration. We inducted the LPS, *L*-Clausenamide, and AKT inhibitor in A549 cells and applied the measurement of ROS accumulation, capase-3 activity with mitochondrial morphology (Figure 9), and DNA fragmentation on early apoptotic cells (Figure 10) [33].

Firstly, we found that the AKT inhibitor prevents the alleviation effect of *L*-Clausenamide on green fluorescence formation, implying its ROS amelioration was negatively affected by the AKT inhibitor (Figure 9a). It is also co-verified by the semi-quantitative analysis (Figure 9b). Next, we applied co-staining of FITC-DEVDG-peptide and HBmito Crimson, the latest mitochondrial inner membrane-specific dye designed for super-resolution microscope observation. Similarly, *L*-Clausenamide treatment alleviates the activity of cleaved caspase-3 and mitochondrial morphological deterioration, which is solidly observed even with a change in dye type (Figure 9c). However, the AKT inhibitor clearly and negatively affects this phenotypic rescue (Figure 9c), which is also supported by the semi-quantitative analysis (Figure 9d). Accordingly, it is clear that the AKT inhibitor disrupted the treatment effect of *L*-Clausenamide on LPS-induced A549 cells.

Next, since ROS activity of cleaved caspase-3 and mitochondrial morphology are classical hallmarks of apoptosis, we aim to directly discuss if the AKT inhibitor disrupts the apoptosis alleviation effect of *L*-Clausenamide. So, again, we applied co-staining of TUNEL and Annexin V-mCherry (Figure 10). Similar to observations of ROS, cleaved caspase-3 activity, and mitochondrial morphology, AKT inhibitor introduction reduces the DNA fragmentation alleviation effect on early apoptotic A549 cells due to *L*-Clausenamide (Figure 10a). Also, the semi-quantitative analysis supported our findings. Since we, by default, assumed the commercially sourced Akt inhibitor with a single and specific target only against the Akt protein, our result did not fully study or eliminate the off-target effect of the Akt inhibitor, or its direct chemical–chemical interaction with *L*-*Clausenamide*, leaving an inevitable limitation on these results.

To summarize, to a certain extent, the AKT inhibitor reduced the improvement effect of *L*-Clausenamide on ROS generation, activation of cleaved caspase-3, mitochondrial morphology changes, and DNA fragmentation in early apoptotic A549 cells induced by LPS.

## 4. Discussion

Acute lung injury (ALI) has been regarded as a commonly recognized clinical disease leading to the consequence of pulmonary function loss and endothelial dysfunction. It is still widespread and is related to the global high incidence rate and mortality, and acute lung injury may also develop into acute respiratory distress syndrome [4]. Acute lung injury may be caused by bacterial infection. In addition, one of the critical factors leading to acute lung injury is lipopolysaccharide (LPS), the primary component of the outer membrane of Gram-negative bacteria. It increases microvascular permeability, causing fluid to enter lung tissue and trigger acute tissue inflammation [34]. LPS-facilitated ALI can mediate the enhancement of ROS levels, resulting in increased oxidative stress and further mitochondrial damage and cell apoptosis [20].

The aim of this study focused on the exploration of the undiscovered molecular mechanism of *L*-Clausenamide about how it regulates oxidative stress to treat LPS-induced ALI, providing a reference for developing new treatment strategies. The ADME result showed that the *L*-Clausenamide meets Lipinski rules and has good pharmacokinetic properties. Moreover, currently evaluating the safety of *L*-Clausenamide, Zhang et al.’s research group has conducted acute and chronic toxicity tests and demonstrated that the no-observed-effect-level dose is 60 mg/kg, which is equivalent to 21 times the clinically recommended dose [35,36,37,38]. More specifically, after 15 min of oral administration, *L*-Clausenamide was detected in the brain, liver, intestine, stomach, ovaries, testes, spleen, kidneys, heart, fat, muscle, lungs, and spinal cord. *L*-Clausenamide was detected in the lung, which validates the ADME prediction and means that *L*-Clausenamide has the potential to improve ALI.

Through network pharmacology analysis, a total of 296 *L*-Clausenamide targets were collected through the PharmMapper database. Subsequently, 5195 targets of acute lung injury and 3007 targets of ROS were collected from DisGeNet and OMIM databases, respectively. Due to the close correlation between the occurrence of oxidative stress and the pathological mechanism of acute lung injury, 152 targets were obtained by taking the intersection of three types of targets as the therapy targets of *L*-Clausenamide in treating acute lung injury by regulating oxidative stress.

Subsequently, PPI analysis was conducted, and the network’s topological parameters, DC, CC, and BC, were further analyzed to screen for core targets. The core target was used for KEGG and GO enrichment analysis. Furthermore, we also analyzed the network’s DC, CC, MNC, and MCC topology parameters. The central targets that met all four top 10 topological parameters were selected as central targets, which were used for molecular docking. Interestingly, we found that AKT1 and caspase-3 (CASP3) appear repeatedly and are very important in both the core target and central target. Caspase-3 is a crucial mediator of apoptosis and a frequently activated death protein, catalyzing the specific cleavage of many key cellular proteins [39]. Also, the cleavage of CASP3 is regulated by phosphorylation of AKT1 in an acute lung injury model [40,41]. In the subsequent experimental verification, further experiments are needed to validate the key role of AKT1 in regulating oxidative stress in the treatment of acute lung injury through *L*-Clausenamide.

Based on GO enrichment analysis, the key biological processes involved in treating acute lung injury by regulating oxidative stress through *L*-Clausenamide may involve positive regulation of programmed cell death (PCD). The PCD includes apoptosis, autophagy, necroptosis, pyroptosis, and ferroptosis [42]. Furthermore, KEGG enrichment analysis also indicated that the key pathway of *L*-Clausenamide in treating acute lung injury by regulating oxidative stress includes apoptosis. Apoptosis is a strictly regulated form of cell death triggered by multiple pathways, with caspase activation playing a central role [43]. Based on the analysis of network pharmacology and GO and KEGG enrichment, we obtained a research hypothesis: *L*-Clausenamide treats LPS-induced acute lung injury by regulating oxidative stress to improve mitochondrial damage and reducing the CASP3-induced apoptosis.

The interactions between *L*-Clausenamide and various key proteins provide significant insights into its potential therapeutic effects for lung injury and related diseases. The molecular docking studies reveal that *L*-Clausenamide exhibits varying degrees of binding affinity across the eight proteins studied. These variations in binding affinity and interaction types reflect the differential impact of *L*-Clausenamide on the functions of the proteins. The hydrophobic interactions, including pi–alkyl, pi–pi, and alkyl bonds, provide structural stability between ligand and receptor, contributing to the overall binding affinity [44]. This accounts for the higher absolute binding affinity values observed with ESR1 and HS90A. Hydrogen bonds in protein–ligand interactions typically indicate conformational changes or modulation of protein function [45]. For AKT1 and *L*-Clausenamide, four hydrogen bonds were formed, including two between Asn231 and the carboxyl and hydroxyl groups of *L*-Clausenamide, and two additional bonds between Lys284 and a different carboxyl and hydroxyl group on *L*-Clausenamide. On the other hand, the amino acid residue Arg341 of CASP3 formed one hydrogen bond with a hydroxyl group in *L*-Clausenamide, which contributes to CASP3 scoring a lower absolute binding affinity compared to AKT1.

The differential binding affinities and interaction types observed between *L*-Clausenamide and these proteins highlight its potential as a modulator of key biological processes. ESR1, HS90A, MMP-9, and AKT1 are all involved in regulating inflammation and tissue remodeling. Increasing the protein level of HS90A and MMP-9 is linked to increased lung injury, while higher ESR1 levels are associated with reduced inflammation [46,47,48,49]. The strong interactions with these proteins suggest that *L*-Clausenamide could be particularly effective in regulating inflammation, stress responses, and cell survival, which are all critical in the context of lung injury. CASP3 controls apoptosis to preserve tissue integrity, ALB maintains plasma oncotic pressure to prevent pulmonary edema and reduce the risk of acute respiratory distress syndrome, and EGFR promotes tissue repair [50,51,52]. Imbalances in CASP3, ALB, and EGFR can lead to lung injury, with low levels of CASP3 and ALB compromising lung function, and dysregulated EGFR potentially causing fibrosis or inflammation. Molecular docking results show that *L*-Clausenamide has moderate binding affinities to these three proteins, suggesting its potential to modulate their activity. Specifically, *L*-Clausenamide interaction with CASP3 through multiple hydrogen bonds may influence apoptosis, helping to maintain the balance necessary for preserving lung tissue integrity. Additionally, *L*-Clausenamide binding to ALB could affect plasma oncotic pressure, while its interaction with EGFR may impact tissue repair processes. However, further study may also focus on the other key proteins, since this study mainly validated the interaction between the AKT1 and *L*-Clausenamide.

Apoptotic cell death is the major player with a central role in inflammation control and facilitates the progression of acute lung injury (ALI) [53,54]. The activation of mitochondrial apoptotic cell death is facilitated through oxidative stimuli response [40]. At the initial stage of ALI, fast ROS accumulation rapidly challenges the antioxidant capacity of the human body and induces apoptosis, followed by recruiting and activating effector proteins like caspase-3 and -7 in programmed cell death execution [54]. Accordingly, scavenging or prevention of ROS accumulation would benefit cell survival under ALI stress [55]. LPS, as the major component of Gram-negative bacterial cells, is widely used as an inducer in ALI cellular modeling [56]. In our study, we found that *L*-Clausenamide, in a concentration-dependent manner, prevents the A549 cell viability loss induced by LPS-stimuli. Considering the role of ROS in the ALI cellular model, following experiments, we found *L*-Clausenamide, at a non-toxic dose, alleviates LPS-induced A549 cellular model of ROS accumulation at both single-cell and bulk culture levels, indicating the mechanism behind it is highly related to ROS scavenging or its consequence prevention.

Mitochondria are the crucial origin of intracellular ROS accumulation and a conspicuous target of ROS attack [57,58]. Mitochondrial damage can affect cellular health and, further, lead to the occurrence of cell apoptosis [59].

As the natural byproduct of oxygen metabolism, ROS is crucial for the maintenance of intracellular signal transduction and homeostasis, facilitating cell survival, differentiation, proliferation, and other activities [60,61]. However, an overwhelming amount of ROS will collapse the cellular and organelle membranes’ phospholipid, stressing the integrity, permeability, normality of potential, and morphology [62,63]. Also, enzymatic reactions of the electron transport chain are also targets of ROS, resulting in electron transport chain chaos, oxidative phosphorylation abnormality, and ATP synthesis disability [64]. All the dysfunction and abnormality of mitochondria above always indicate the hallmark of “no return timepoint”, leading to the programmed cell death and maturation of effector proteins like caspase-3 [65,66]. Caspase-3, a well-studied caspase executor protein, takes responsibility for the collapse of multiple-target proteins by recognition of the DEVDG sequence at the death execution phase of apoptosis [67,68]. However, before this irreversible progress and at a prior stage, ROS-mediated oxidative stress insults the morphological normality and integrity of the mitochondrial membrane, and consequently facilitates permeability compromise alongside the abnormality of proton motive force, which is reflected by mitochondrial membrane potential [69]. Moreover, the oxidative stress will also activate endogenous caspase pathways by activating the caspase-3 [70]. Therefore, prevention of these early molecular events is beneficial for cellular survival [71]. Meanwhile, activation of caspase-3 in cleaved form in the regulation of apoptotic programmed cell death is regulated via the AKT1 phosphorylation amount [40]. AKT1 phosphorylation levels can ameliorate oxidative stress and mitochondrial-related damage and apoptosis for cell survival [72,73]. One of the mechanisms of phosphorylation of AKT1 is to promote cell survival and inhibit apoptosis through inhibiting caspase-3 [74]. More specifically, Goyal et al.’s study showed that the phosphorylation of AKT can inhibit the cleavage of caspase-3 and apoptosis to promote cell survival [75,76].

In our study, we observed the LPS-induced ALI A549 cellular model with classical apoptotic phenotypes like ROS accumulation, cellular and mitochondrial membrane potential decrease, ATP loss, and enhancement in cleaved caspase-3 activity alongside mitochondrial morphology abnormality. However, in a concentration-dependent manner, *L*-Clausenamide significantly rescues or alleviates the phenotypic changes above, indicated through suppression in caspase-3-related mitochondrial apoptosis initiation. Moreover, we also performed the SPR analysis and Western blot analysis to further study the molecular mechanism of *L*-Clausenamide. The combination of SPR and molecular docking is a scientific approach to studying the interaction between the ligand and receptors [77]. And our molecular docking results showed a good binding affinity (−6.9 kcal/mol) between AKT1 and *L*-Clausenamide, lower than −5 kcal/mol [78]. Similarly, the results of SPR analysis also validated this docking result and showed that ligand (*L*-Clausenamide) and receptor (AKT1) has a relatively low Kd value (10.3 μM) and a clear dynamic figure (Figure 7b) including the binding curve and dissociation curve [79].

Indeed, the cleaved caspase-3 can degrade Akt1 and inhibit its phosphorylation, and *L*-Clausenamide may interact with caspase-3 to improve the phenotype. So, we also performed a supplementary experiment using the AKT1 inhibitor to prove that *L*-Clausenamide achieves the above bioactivity through targeting AKT1 instead of caspase-3. And the result showed that the Akt inhibitor directly reduces the improvement effect of *L*-Clausenamide on ROS generation, activation of cleaved caspase-3, mitochondrial morphology changes, and DNA fragmentation in early apoptotic A549 cells induced by LPS. To summarize, the above result showed that the *L*-Clausenamide can directly bind to AKT1 instead of caspase-3 and further regulate the phosphorylation of AKT, which can inhibit the cleavage of caspase-3 and alleviate the acute lung injury.

**Figure 11:**
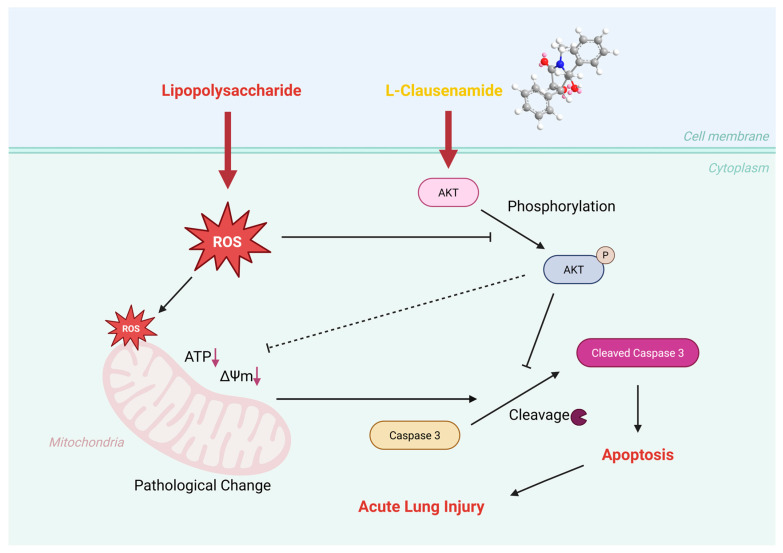
The molecular mechanism of *L*-Clausenamide improving the acute lung injury (ALI) mediated by lipopolysaccharide (LPS).

Taken together, *L*-Clausenamide can alleviate the LPS-induced A549 cell viability (ALI cellular model) decrease and may target AKT1 through alleviation of mitochondrial abnormality and apoptosis. Our results proved the developmental potential of *L*-Clausenamide in the prevention of ALI in early stages through mitochondrial apoptosis inhibition, and the molecular mechanism of *L*-Clausenamide, improving the LPS-induced ALI, was concluded in Figure 11. However, further animal studies should be conducted to study its effect on acute lung injury in vivo, which uses some specific transgenic mice to further validate the regulating effect of *L*-Clausenamide in AKT1. In addition, the other mechanism of *L*-Clausenamide should also be studied, since the induction of the AKT inhibitor does not fully abrogate Clausenamide’s effects (Figure 9 and Figure 10), and there are still some other potential targets worth further study, besides AKT1 (Figure 2 and Figure 3). Meanwhile, since our results did not eliminate the off-target effect of the AKT inhibitor or its direct effect on *L*-Clausenamide, there is still a limitation of this study, which may lead to the effect between the AKT inhibitor and *L*-Clausenamide not being fully understood, and requires further study in the future for a more comprehensive understanding.

## 5. Conclusions

This study elucidated the improving effect of *L*-Clausenamide in treating LPS-induced acute lung injury and its underlying molecular mechanisms. A total of 152 anti-acute lung injury targets, 61 core targets, and 8 central targets were identified. Combined with the PPI analysis, AKT1 is an important target of *L*-Clausenamide’s improving effect in acute lung injury. Based on the GO and KEGG enrichment analysis, the apoptosis pathway is involved in the alleviating effect of *L*-Clausenamide. *L*-Clausenamide alleviates LPS-induced ALI by inhibiting caspase-3 cleavage activity and normalizing intracellular ATP level, cellular and mitochondrial membrane potential, and mitochondrial morphology. Moreover, *L*-Clausenamide can target AKT1 and regulate its phosphorylation to inhibit the cleavage of caspase-3. These findings provide a primary basis for a better understanding of how *L*-Clausenamide provides benefits in LPS-induced ALI and lay a foundation for the development of new potential drug-lead phytochemicals curing ALI.

## Figures and Tables

**Table 1:** The ADME Analysis of *L*-Clausenamide.

Compound Name		*L*-Clausenamide
PubChem ID		9904294
Lipinski rules	Molecular weight (<500)	297.35
	H-bond acceptors (<10)	3
	H-bond donors (<5)	2
	MLogP (Log Po/w) (<4.15)	1.60
	Lipinski’s violations (<1)	0
Blood–Brain Barrier (BBB) permeant		Yes
Gastrointestinal (GI) absorption		High
Bioavailability Score (>0.1)		0.55
Log Kp (skin permeation)		−7.02 cm/s

## Data Availability

The datasets used and analyzed during the current study are available from the corresponding author on reasonable request.

## Supplementary Materials

The following supporting information can be downloaded at: https://www.mdpi.com/article/10.3390/biology14070836/s1, Supplementary Figure S1. The original figure of Figure 5a; Supplementary Figure S2. The original figure of Figure 5d; Supplementary Figure S3. The original figure of Figure 6a; Supplementary Figure S4. The original figure of Figure 6d; Supplementary Figure S5. The original figure of Figure 7a; Supplementary Figure S6. The original figure of Figure 9a; Supplementary Figure S7. The original figure of Figure 9c; Supplementary Figure S8. The original figure of Figure 10a.

## Abbreviations

AKT1	RAC-alpha serine/threonine-protein kinase
ALB	serum albumin
ALI	acute lung injury
Arg	arginine
ATP	Adenosine 5′-triphosphate
BC	betweenness centrality
CASP3	Caspase-3
CC	closeness centrality
CC	cellular components
Cys	cystine
em	emission
DC	degree centrality
DEVDG	Aspartate-Glutamate-Valine-Aspartate-Glycine
DiBaC4	bis-(1,3-dibutylbarbituric acid) trimethine oxonol
DNA	deoxyriboNucleic Acid
EGFR	epidermal growth factor receptor
ESR1	estrogen receptors
FITC	Fluorescein Isothiocyanate
GO	Gene Ontology
HSP90AA1	heat shock protein 90 alpha family class A member 1
JC-1	5,5′,6,6′-tetrachloro-1,1′,3,3′-tetraethylbenzimi-dazolylcarbocyanine iodide
KEGG	Kyoto Encyclopedia of Genes and Genomes
LPS	Lipopolysaccharide
MCC	Maximal Clique Centrality
MF	molecular functions
MMP-9	matrix metalloproteinase-9
MNC	Maximum Neighborhood Component
nm	nanometer
OMIM	Online Mendelian Inheritance in Man
PCD	programmed cell death
PMF	proton motive force
PPI	protein-protein interaction
RLU	relative luminescent unit
RFU	relative fluorescent unit
ROS	reactive oxygen species
RMSD	root mean square deviation plot
RMSF	root mean square fluctuation plot
Rg	radius of gyration plot
SPR	Surface Plasmon Resonance
SASA	solvent accessible surface area plot
SIM	Structured Illumination Microscopy
SRC	proto-oncogene tyrosine-protein kinase
2D	2-Dimensional
3D	3-Dimensional
