# Supplementary material for: Revealing the Improving Effect and Molecular Mechanism of L-Clausenamide in Combating the Acute Lung Injury: Insights from Network Pharmacology, Molecular Docking, and In Vitro Validation
Source: Biology (Basel). 2025 Jul 9;14(7):836. doi: 10.3390/biology14070836 (PMC12292302; doi:10.3390/biology14070836)
Supplement: Supplementary file 1 [file biology-14-00836-s001.zip › biology-3630866-supplementary.pdf]

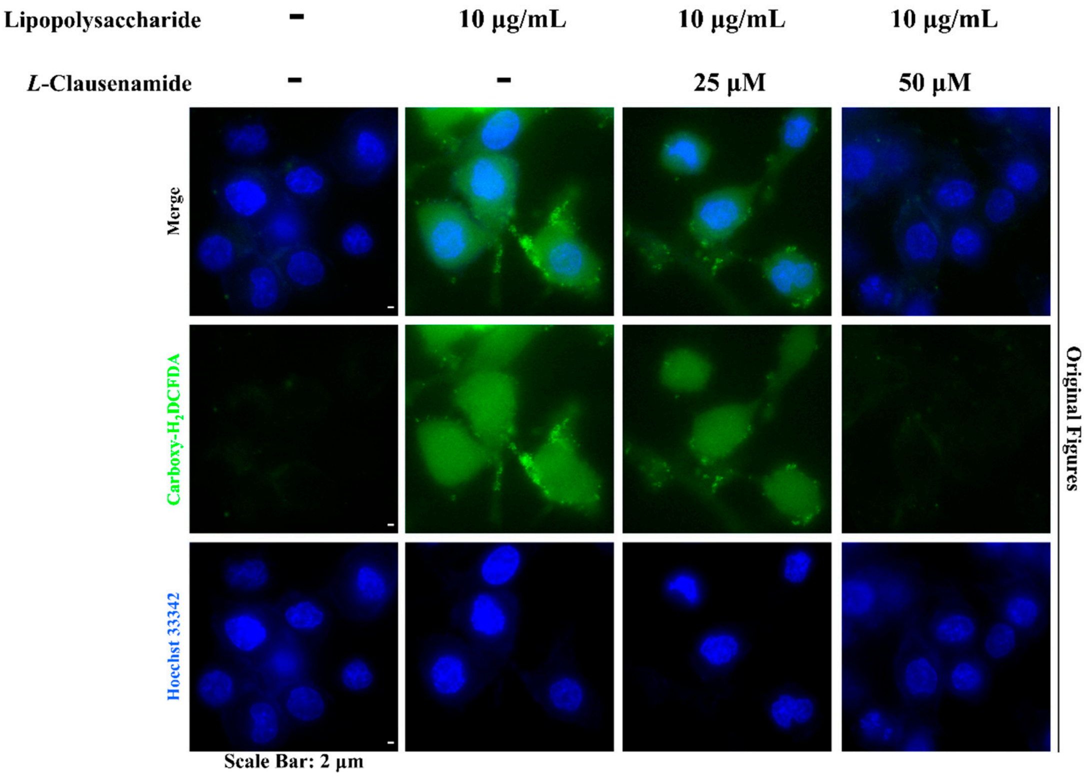

Supplementary figure S1. The original figure of figure 5a.

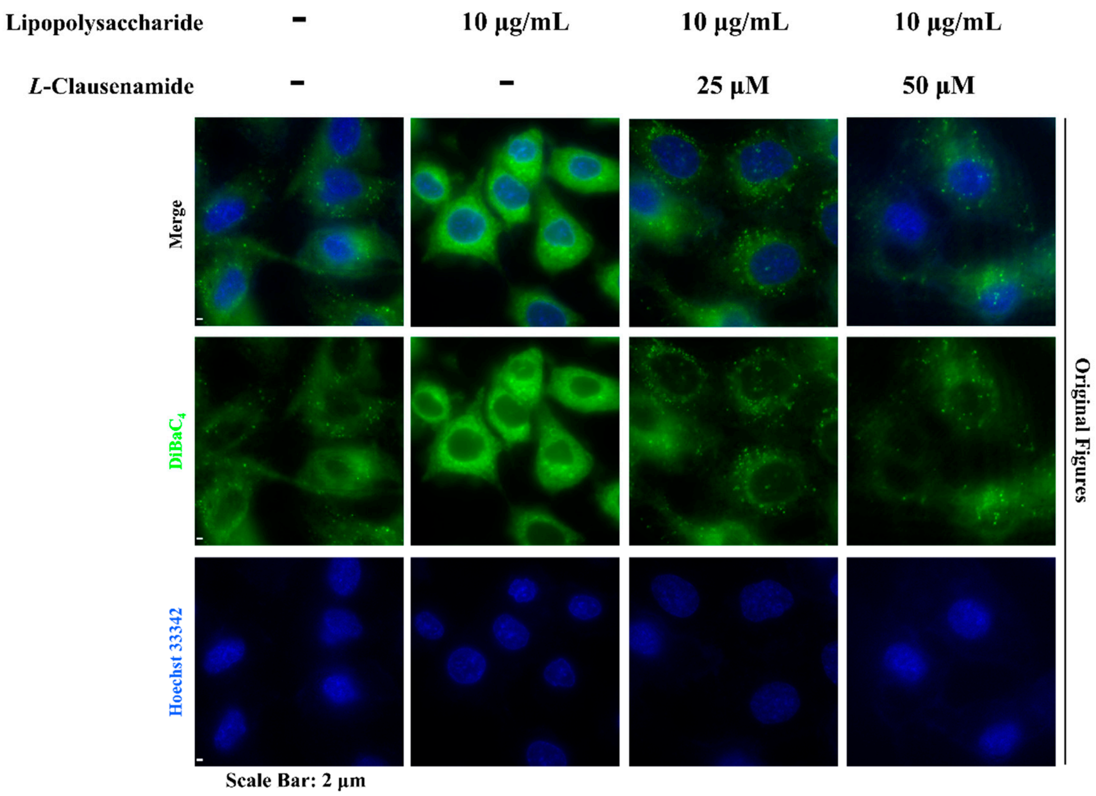

Supplementary figure S2. The original figure of figure 5d.

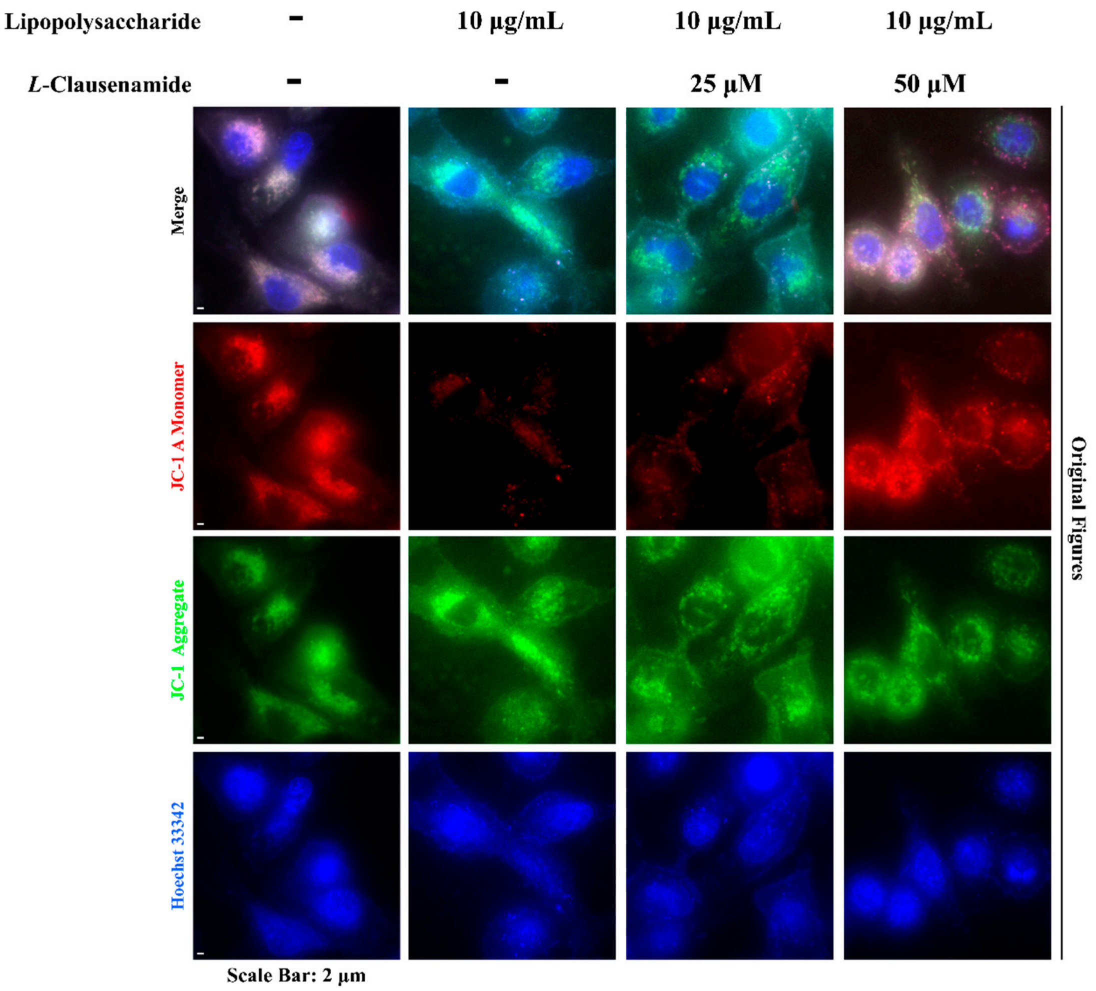

Supplementary figure S3. The original figure of figure 6a.

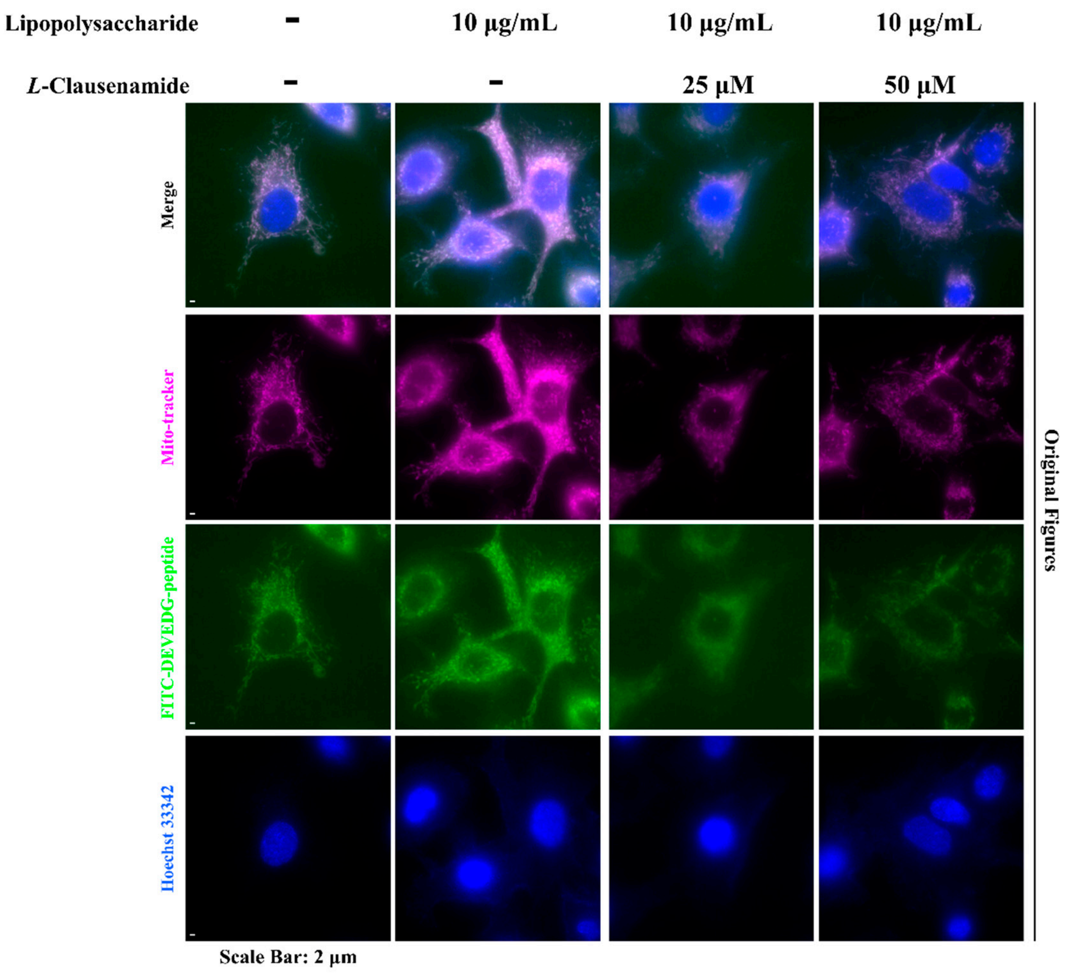

Supplementary figure S4. The original figure of figure 6d.

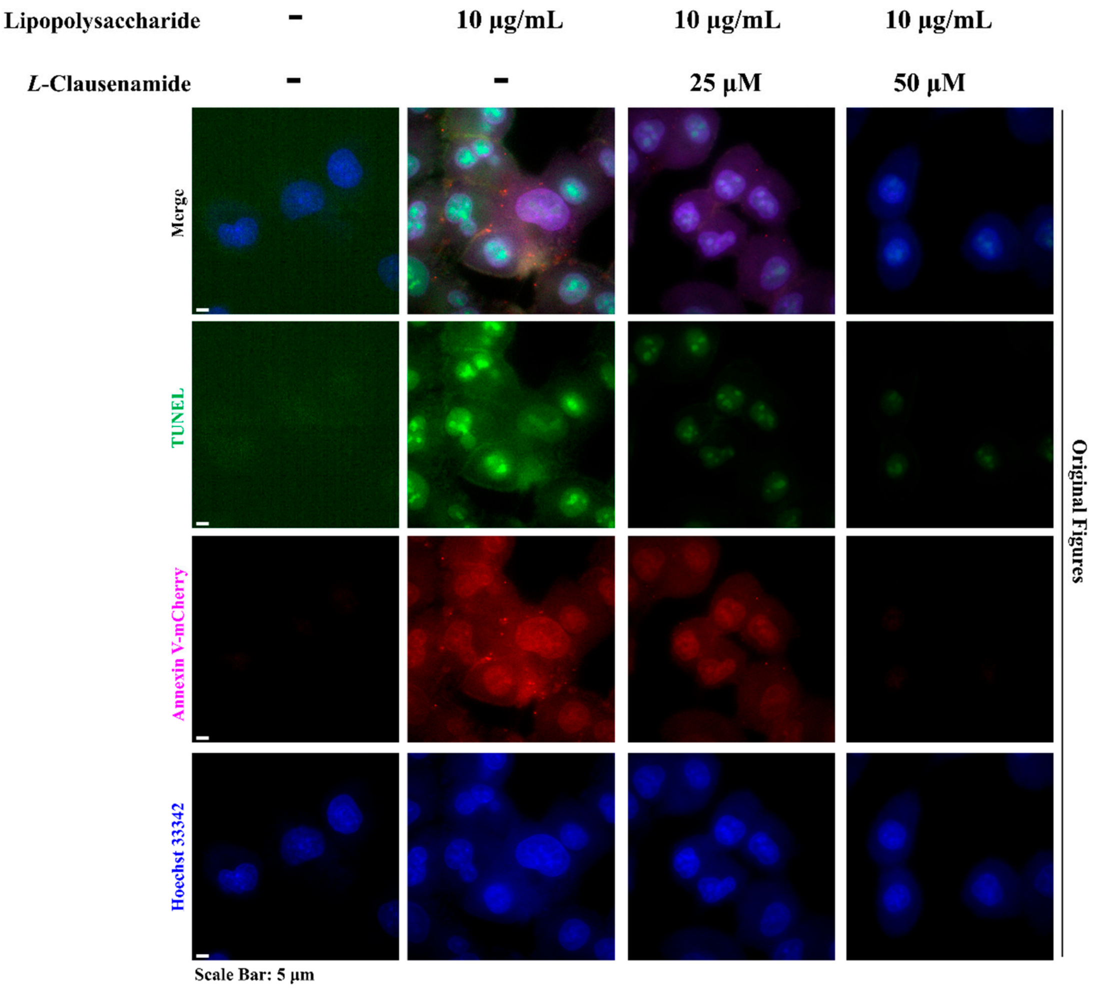

Supplementary figure S5. The original figure of figure 7a.

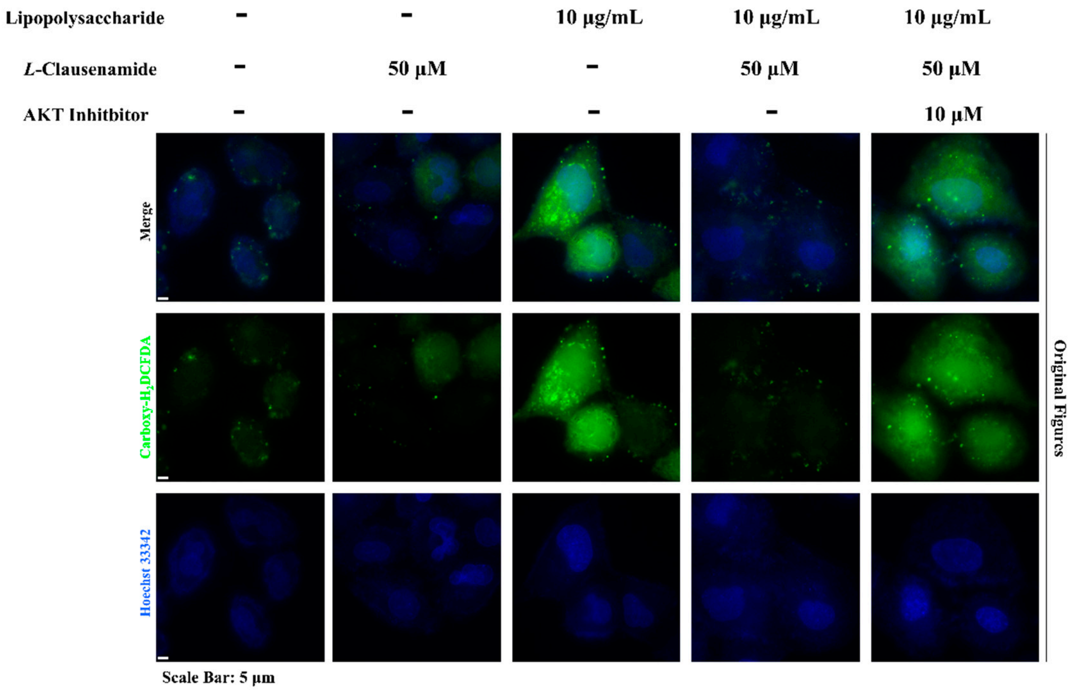

Supplementary figure S6. The original figure of figure 9a.

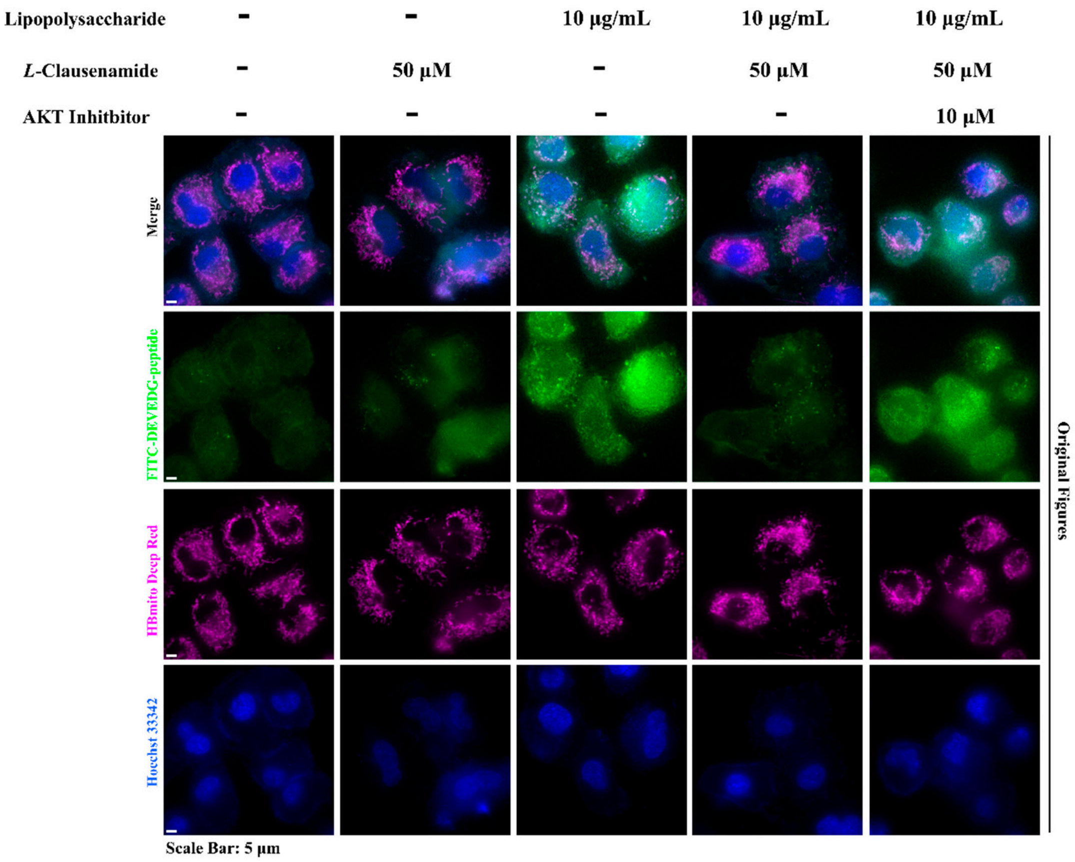

Supplementary figure S7. The original figure of figure 9c.

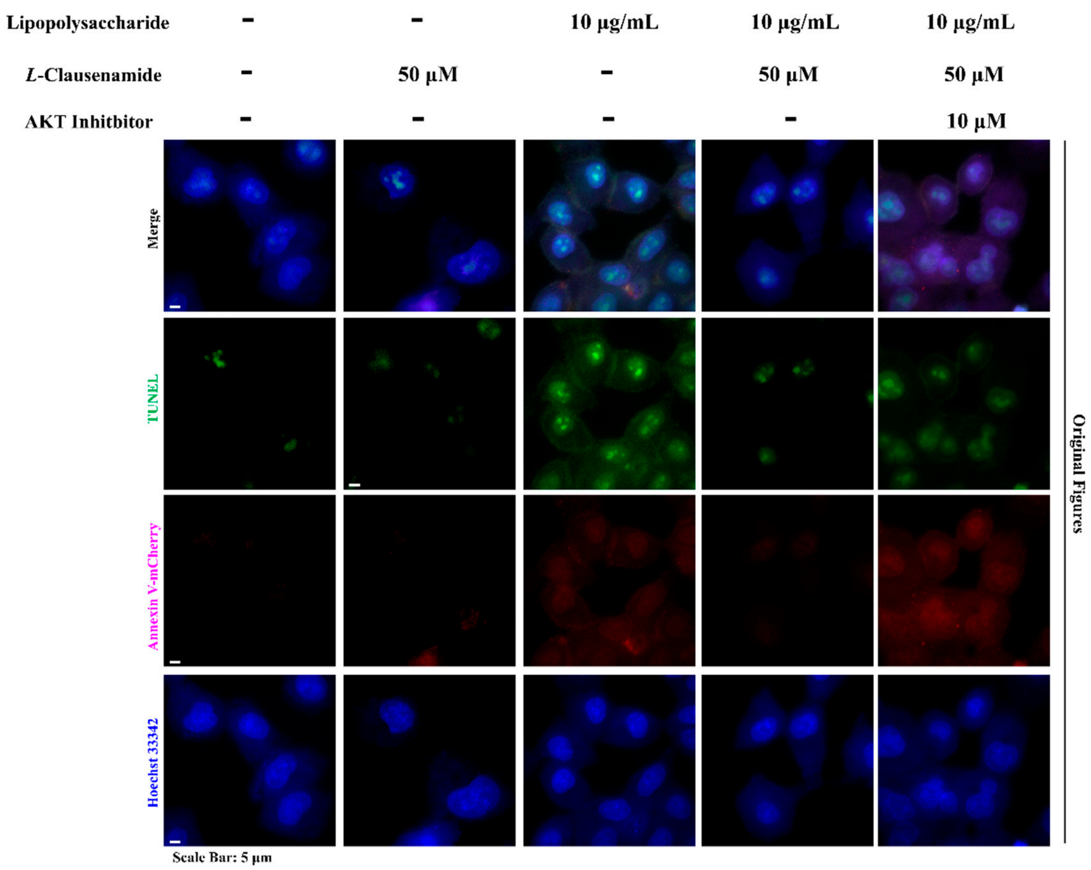

Supplementary figure S8. The original figure of figure 10a.
