# Supplementary figures and images for: Revealing the Improving Effect and Molecular Mechanism of L-Clausenamide in Combating the Acute Lung Injury: Insights from Network Pharmacology, Molecular Docking, and In Vitro Validation
Source: Biology (Basel). 2025 Jul 9;14(7):836. doi: 10.3390/biology14070836 (PMC12292302; doi:10.3390/biology14070836)

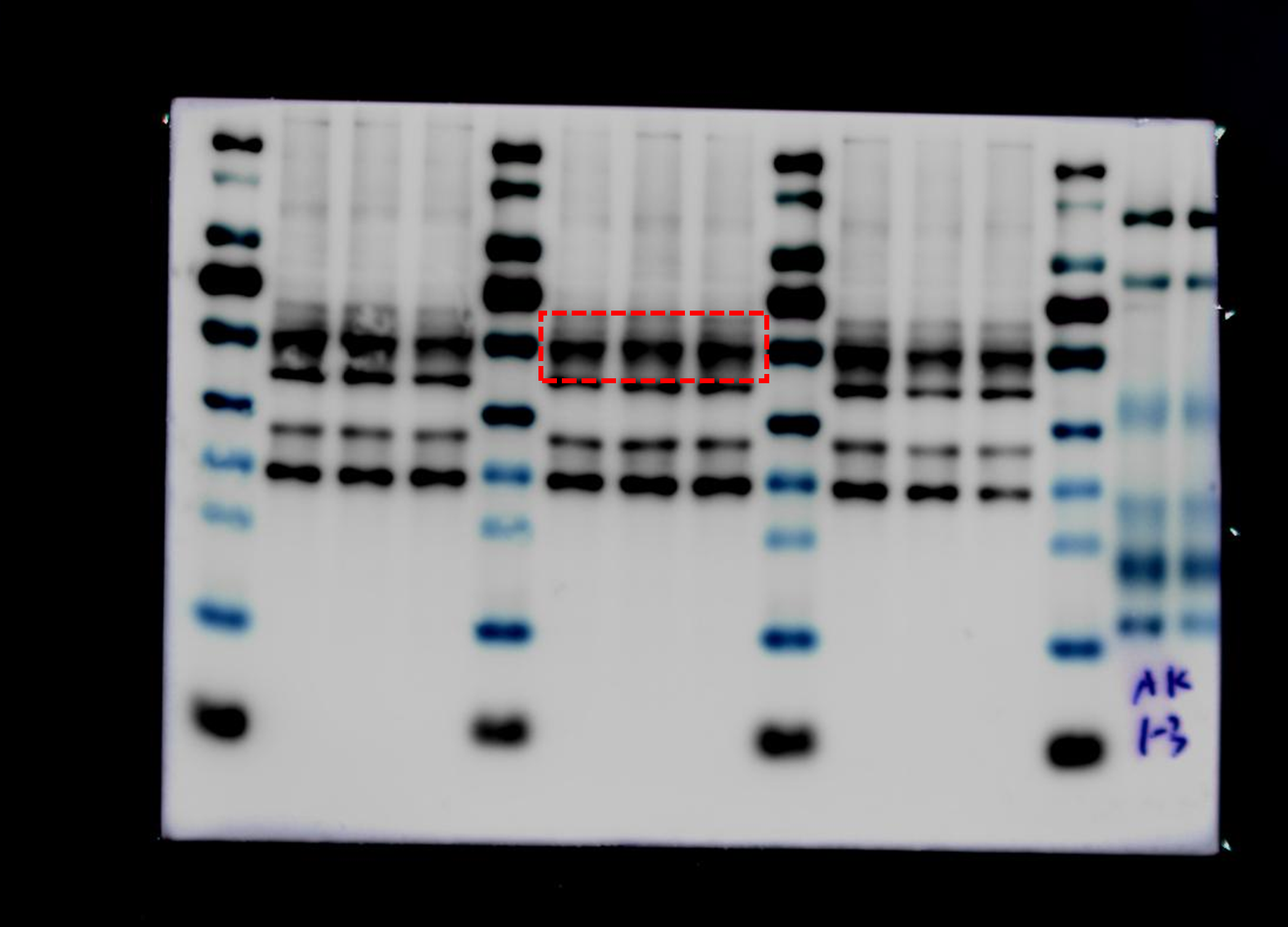

Supplement: Supplementary file 1 [file biology-14-00836-s001.zip › Figure 8C-AKT.PNG]

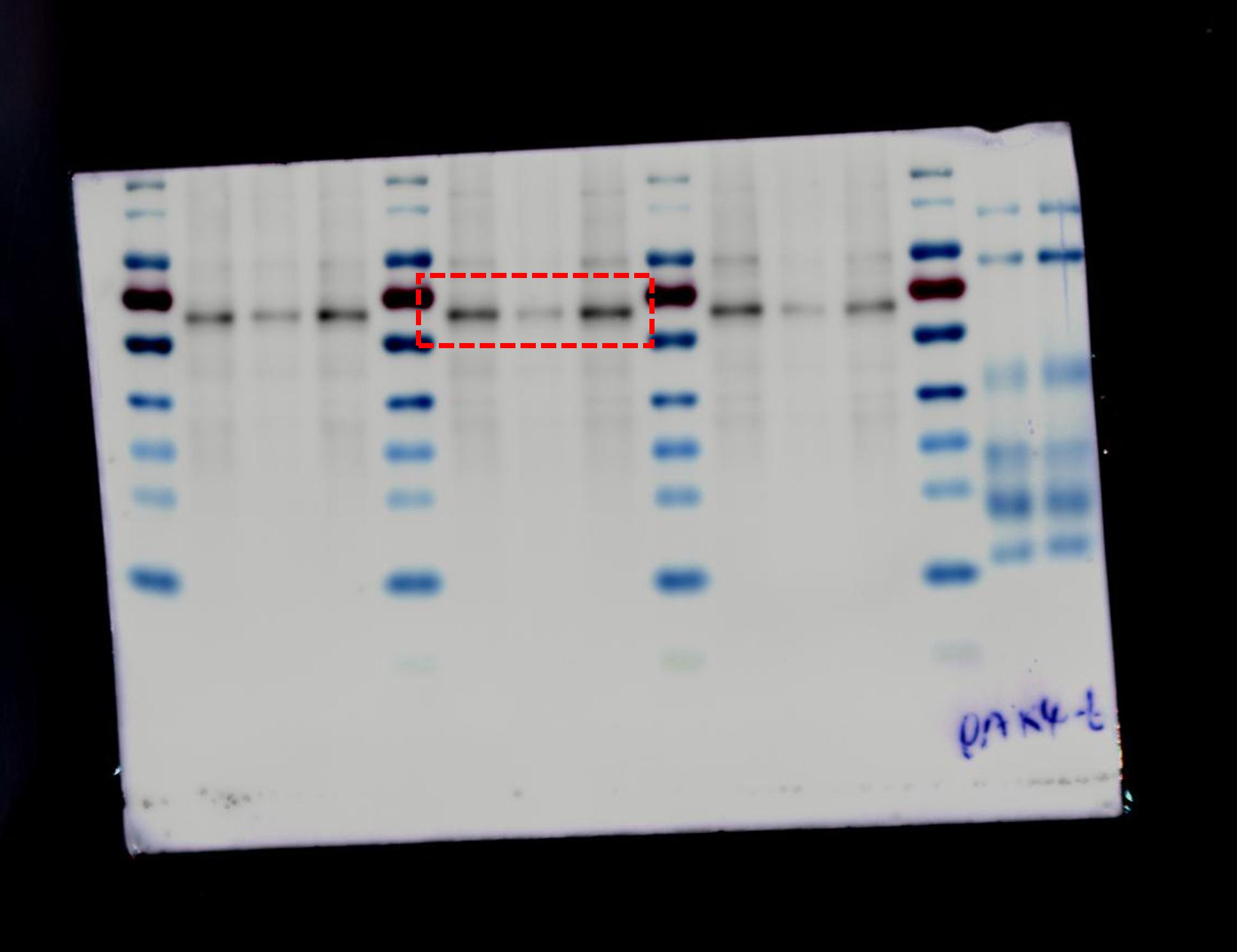

Supplement: Supplementary file 1 [file biology-14-00836-s001.zip › Figure 8C-p-AKT.PNG]

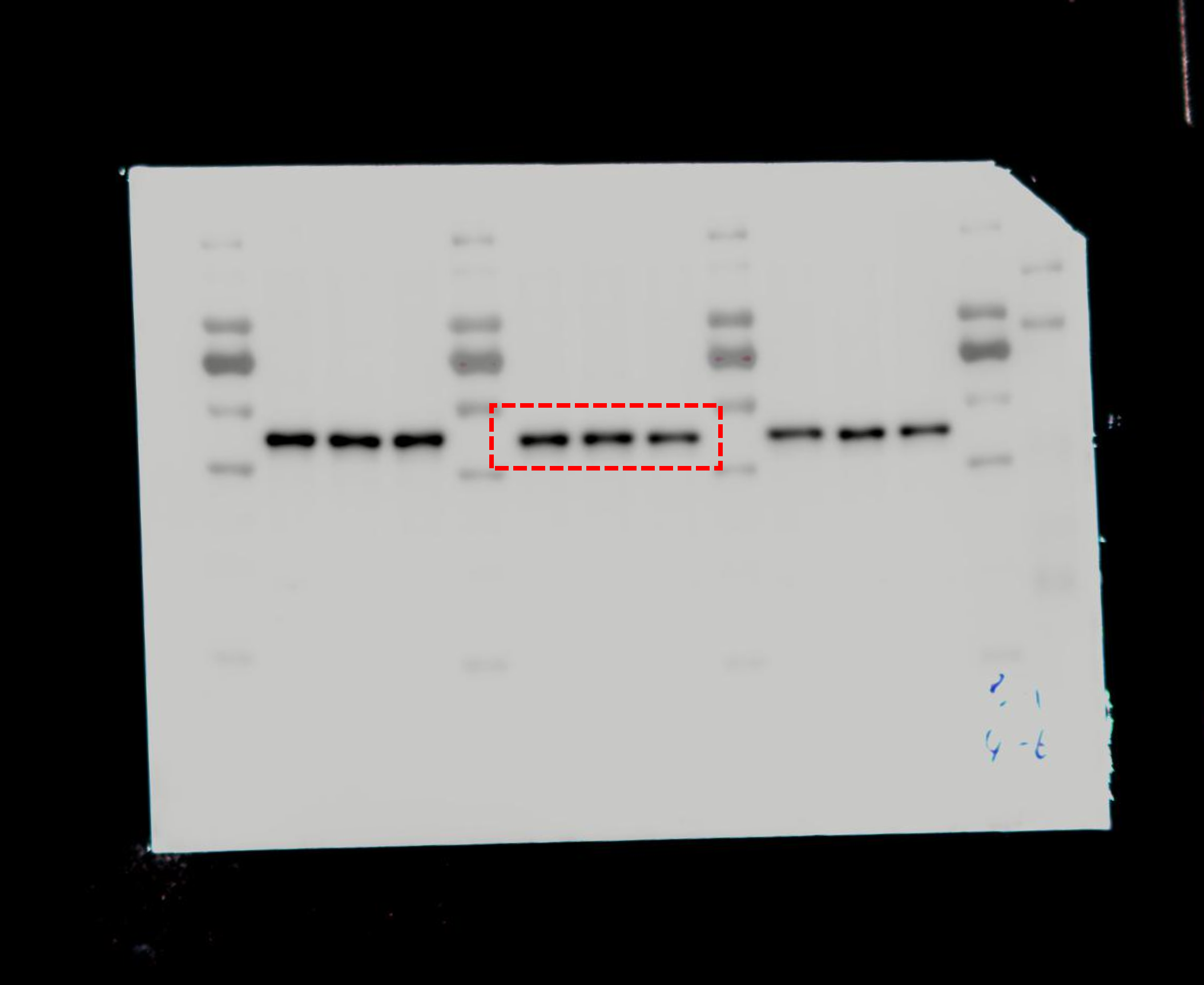

Supplement: Supplementary file 1 [file biology-14-00836-s001.zip › Figure 8C. b-actin.PNG]

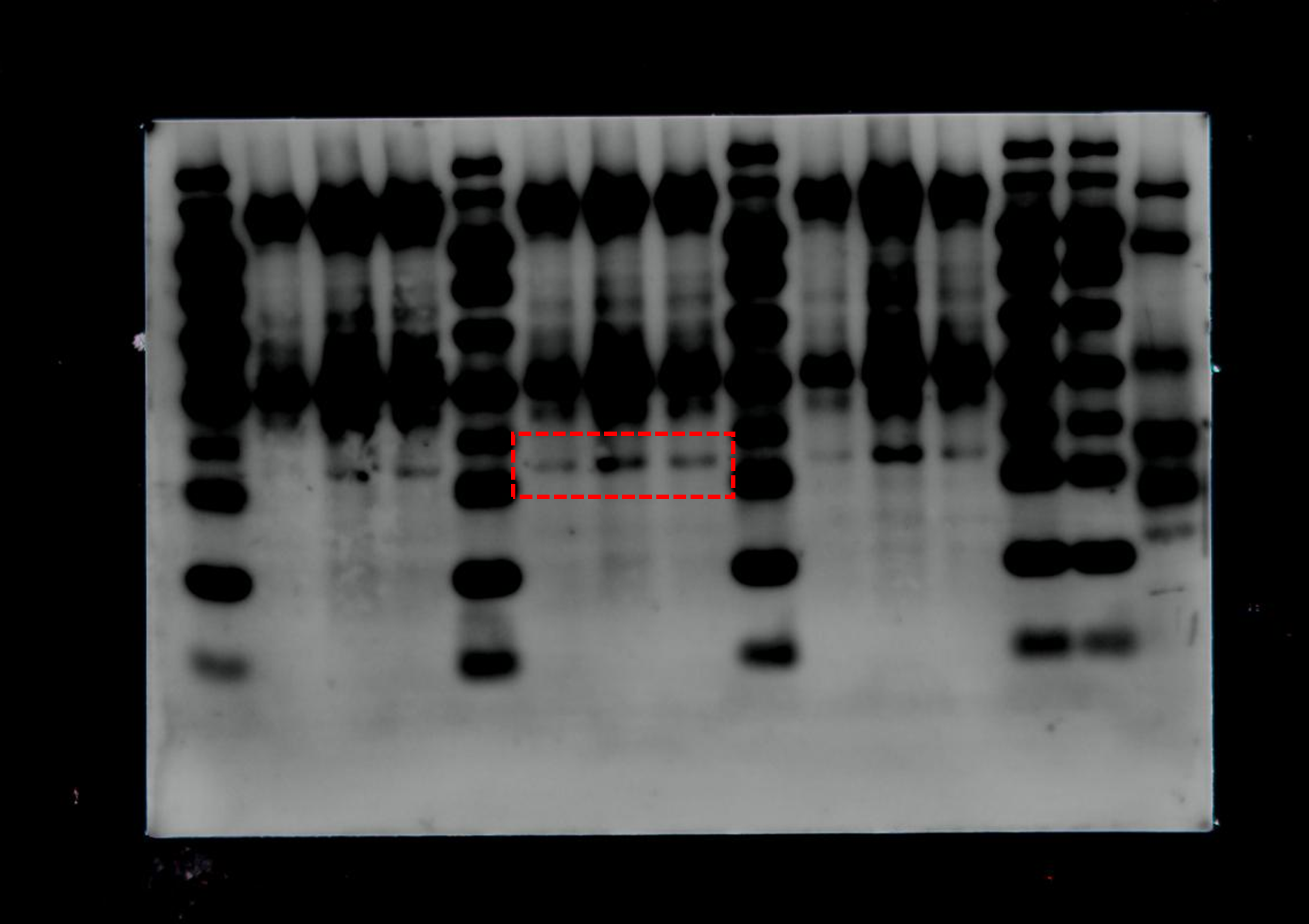

Supplement: Supplementary file 1 [file biology-14-00836-s001.zip › Figure 8C. Cleaved Caspase 3.PNG]
